# Characterization of Unexplored Deadwood Mycobiome in Highly Diverse Subtropical Forests Using Culture-independent Molecular Technique

**DOI:** 10.3389/fmicb.2017.00574

**Published:** 2017-04-19

**Authors:** Witoon Purahong, Katherina A. Pietsch, Guillaume Lentendu, Ricardo Schöps, Helge Bruelheide, Christian Wirth, François Buscot, Tesfaye Wubet

**Affiliations:** ^1^Department of Soil Ecology, UFZ-Helmholtz Centre for Environmental ResearchHalle, Germany; ^2^Department of Systematic Botany and Functional Biodiversity, University of LeipzigLeipzig, Germany; ^3^Department of Ecology, Technical University of KaiserslauternKaiserslautern, Germany; ^4^Institute of Biology/Geobotany and Botanical Garden, Martin Luther University Halle-WittenbergHalle, Germany; ^5^German Centre for Integrative Biodiversity Research (iDiv)Leipzig, Germany

**Keywords:** fungal diversity, subtropical forest, BEF China, pyrosequencing, ecosystem processes, wood decomposition, abiotic factor, biotic factor

## Abstract

The deadwood mycobiome, also known as wood-inhabiting fungi (WIF), are among the key players in wood decomposition, having a large impact on nutrient cycling in forest soils. However, our knowledge of WIF richness and distribution patterns in different forest biomes is limited. Here, we used pyrotag sequencing of the fungal internal transcribed spacer (ITS2) region to characterize the deadwood mycobiome of two tree species with greatly different wood characteristics (*Schima superba* and *Pinus massoniana*) in a Chinese subtropical forest ecosystem. Specifically, we tested (i) the effects of tree species and wood quality properties on WIF OTU richness and community composition; (ii) the role of biotic and abiotic factors in shaping the WIF communities; and (iii) the relationship between WIF OTU richness, community composition and decomposition rates. Due to different wood chemical properties, we hypothesized that the WIF communities derived from the two tree species would be correlated differently with biotic and abiotic factors. Our results show that deadwood in subtropical forests harbors diverse fungal communities comprising six ecological functional groups. We found interesting colonization patterns for this subtropical biome, where *Resinicium* spp. were highly detected in both broadleaved and coniferous deadwood. In addition, the members of Xylariales were frequently found in *Schima*. The two deadwood species differed significantly in WIF OTU richness (*Pinus* > *Schima*) and community composition (*P* < 0.001). Variations in WIF community composition of both tree species were significantly explained by wood pH and ecological factors (biotic: deadwood species, basal area and abiotic: soil pH), but the WIF communities derived from each tree species correlated differently with abiotic factors. Interestingly, we found that deadwood decomposition rate significantly correlated with WIF communities and negatively correlated with WIF OTU richness. We conclude that the pattern of WIF OTU richness and community composition are controlled by multiple interacting biotic and abiotic factors. Overall, our study provides an in-depth picture of the deadwood mycobiome in this subtropical forest. Furthermore, by comparing our results to results from temperate and boreal forests we contribute to a better understanding of patterns of WIF communities across different biomes and geographic locations.

## Introduction

Deadwood is an important structural component of natural forest ecosystems and harbors up to one million wood-inhabiting species worldwide (Stokland et al., 2012). These species belong to highly diverse taxonomic groups, including bacteria, fungi, invertebrate and vertebrate animals (Stokland et al., 2012; Hoppe et al., 2014, 2015; Purahong et al., 2014a,b; Müller et al., 2015). Besides its importance as habitat for life within forests, deadwood also plays important roles in biogeochemical processes and ecosystem functioning as an energy and nutrient source (Hodge and Peterken, 1998). Deadwood stores energy in various forms of diverse complex polymeric organic compounds of carbohydrates (cellulose and hemicellulose) and also aromatic substances (lignin), and this energy can be released via various decomposition processes (Hodge and Peterken, 1998). Compared to other substrates, deadwood is known as being recalcitrant with regard to decomposition due to its high lignin content (Floudas et al., 2012). Fungi are the major components of the microbiome that substantially decompose all polymeric carbon compounds, including lignin (Rajala et al., 2010, 2012). Therefore, wood-inhabiting fungi (WIF) or the wood mycobiome are among the key players in wood decomposition with a large significant impact on the nutrient cycling and carbon dynamics of forests (Rajala et al., 2012).

Species richness and distribution patterns of microorganisms provide important insights into their roles for ecosystem functioning and stability (Kubartová et al., 2012; Purahong et al., 2016a). In this respect, our knowledge about WIF is only fragmentary, due to the low resolution of fungal community detection methods, erratically distributed study sites and incomplete coverage of different biomes, especially the subtropical one (Kubartová et al., 2012; Seibold et al., 2015; Hoppe et al., 2016). Most studies on WIF diversity have relied on sporocarp surveys or mycelia isolations, which do not allow total community evaluation (Kubartová et al., 2012). Recent studies using next generation sequencing have greatly improved our understanding of the distribution patterns of WIF (Kubartová et al., 2012; Ovaskainen et al., 2013; Hiscox et al., 2015; Ottosson et al., 2015; Yamashita et al., 2015; Hoppe et al., 2016). Molecular studies of wood-inhabiting fungal diversity have so far been restricted to temperate and boreal forests in Europe, which characterized low plant diversity levels (i.e., tree species richness ranging from one to six species) (Kubartová et al., 2012; Ottosson et al., 2015; Hoppe et al., 2016). Thus, the effect of high plant diversity on WIF diversity and community composition are yet not well understood.

Significant drivers for WIF OTU richness and distribution patterns include abiotic factors (wood physicochemical properties) of the decomposing deadwood such as wood density, size, water content, pH, C, N, C: N, lignin content (Rajala et al., 2012; Purahong et al., 2014a,b; Hoppe et al., 2016) as well as with the fungal assemblage history (Fukami et al., 2010; Dickie et al., 2012; Hiscox et al., 2015), interaction between specific WIF (Hoppe et al., 2016), and identities and abundances of saproxylic insects (Jacobs and Work, 2012; Floren et al., 2015), the latter three being the biotic component. While these abiotic factors are species specific, they also change during the course of decomposition (Baldrian et al., 2016). Saproxylic insects can act as vectors for WIF but, they are also known to feed on fungal hypha (Stokland et al., 2012). Additionally, WIF OTU richness and distribution patterns are influenced by plot-level parameters, such as deadwood quantity and quality, forest structure and management, as well as micro-climate (Purahong et al., 2014a,b). These drivers have been identified from temperate and boreal forests. Unfortunately, similar experimental studies in subtropical and tropical forests are limited (Seibold et al., 2015). Previous studies from tropical and subtropical forests have revealed that wood physical properties and the abundance of tree species influence the community composition of some groups of WIF (Gilbert and Sousa, 2002; Ferrer and Gilbert, 2003). In tropical and subtropical forests, a high diversity of tree species can reduce chances for WIF to colonize the specific tree species since the probability of successful colonization decreases as tree species become increasingly rare (May, 1991; Lindblad, 2000). However, tree species specificity or preferences of WIF are also found in tropical forests when species diversity is low and the abundance of individual tree species is high (Gilbert and Sousa, 2002; Ferrer and Gilbert, 2003). Nevertheless, it remains unclear what drives tree species specificity of different functional groups of WIF in highly diverse tropical or subtropical forest ecosystems.

Deadwood-level parameters have generally received more attention than plot-level ones. Plot-level parameters, including plant community composition, stage of forest succession and elevation have been shown to be related to soil and ectomycorrhizal (ECM) fungal communities in subtropical forests (Wu et al., 2013; Geml et al., 2014). Strong links have also been reported between plant and fungal diversity from tropical forests (Peay et al., 2013). Elevation significantly affects soil fungal community composition in tropical and subtropical mountain forests (Geml et al., 2014). Yet, no similar study is available with respect to WIF. In particular, knowledge on the link between plot-scale plant diversity and single as well as multispecies derived deadwood inhabiting mycobiome is missing.

In this study we conducted a deadwood decomposition experiment using *Pinus massoniana* Lamb. and *Schima superba* Gardn. et Champ. in their native, highly diverse mixed evergreen subtropical forest located along an elevational gradient in South-East China. The two species were selected because they are among the most common species in this forest ecosystem, ranking second and third in total basal area after *Castanopsis eyrie* (Barrufol et al., 2013). *P. massoniana* as well as *S. superba* are native to this region and are both commercially used species which are widely planted in this area. The species differ greatly in their wood characteristics (anatomy and chemical compositions) and mycorrhizal status, traits known as good predictors of wood decomposability (Weedon et al., 2009; Pietsch et al., 2014; Hoppe et al., 2016). To our knowledge, this study is the first comparative analysis of the deadwood mycobiome in subtropical forests.

Accordingly our main aim was to shed light on the knowledge gap on how plant diversity, community composition and elevation affect the richness and community composition of the deadwood mycobiome. The role of Xylobiont insects was investigated through an exclusion treatment using two mesh sizes where samples were placed along a gradient of forest succession and tree diversity. The fungal community was assessed using pyrotag sequencing of the fungal internal transcribed spacer (ITS) region of the rRNA gene. We tested: (i) the effects of tree species and wood quality properties on WIF OTU richness and community composition; (ii) the role of biotic (i.e., tree species diversity, stage of forest succession, insect exclusion) and abiotic factors in shaping the WIF communities; and (iii) the relationship between WIF OTU richness, community composition and decomposition rates. In this subtropical forest, we expected non-specific and diverse WIF communities (relative to temperate forest) composed of different fungal functional groups along the forest successional gradient. Furthermore, due to different wood chemical properties, we hypothesized that the WIF communities derived from the two tree species would be correlated differently with biotic and abiotic factors. In line with earlier studies in temperate forests, we expected strong competition among the WIF in this biome due to the high fungal richness and consequently a negative or non-significant correlation between fungal richness and wood decomposition rate (Hoppe et al., 2016). We assume that in highly diverse WIF communities, there is a high degree of interaction among co-occurring WIF species, which may result in a switch of the energy investment into competition rather than on producing wood-degrading enzymes (Fukami et al., 2010; Dickie et al., 2012; Hoppe et al., 2016). Due to common functional redundancy within the mycobiome we expected limited relationships between WIF community composition and wood decomposition rate (Hoppe et al., 2016). We tested the effect of interaction between specific WIF OTUs on wood decomposition rates.

## Materials and Methods

### Study Site

This experiment was conducted in the Gutianshan National Nature Reserve (GNNR, 81 km^2^, 29°08′ – 29°17′ N, 118°27′ – 118°11′ E) located in Zhejiang province, South-East China, as part of the Biodiversity-Ecosystem Functioning (BEF-China) project. The vegetation is a moist mixed subtropical broadleaved forest (Bruelheide et al., 2011). The mean annual temperature is 15.1°C with a minimum of -6.8°C recorded in January and a maximum of 38.1°C in July. The GNNR represents a gradient of successional stages of secondary forests (20–190 years) that has not been subjected to cutting or other management since the beginning of the 1990s. In addition, conifers (mainly *Cunninghamia lanceolata* (Lamb.) Hook. and *P. massoniana*) have been planted and reproduce all over the GNNR (Bruelheide et al., 2011). We have followed the classification of successional stages of forests from Bruelheide et al. (2011) and the forest plots used in this experiment follow the natural succession in subtropical forest. The observational gradient consisted of 27 (30 m × 30 m) permanent study plots located within different forest successional stages (1, < 20 year; 2, < 40 year; 3, < 60 year; 4, < 80 year; 5, ≥ 80 year), elevations (from 251 to 903 m a.s.l.) and levels of tree and shrub species richness (147 species in total, ranging from 25 to 69 species per 900 m^2^ plot). Detailed information pertaining to these forest plots is summarized in Plot description (Supplementary Material) and details have been presented elsewhere (Bruelheide et al., 2011; Staab et al., 2014; Schuldt et al., 2015).

### Plot-based Data

To understand how plot-related biotic and abiotic factors shape the WIF communities, we used plot-based data collected during plot setup in 2008. Detailed methods of data collection have been described elsewhere (Bruelheide et al., 2011). Explanations of all plot-level biotic and abiotic factors used are shown in the Supplementary Material. We tested for collinearity between all plot-related factors using Spearman’s rank correlation (ρ). When two or more factors were correlated (ρ > 0.70 or ρ < -0.70 and *P* < 0.01), only the factor that yielded the highest correlations with other factors was retained for further analysis (Spearman’s rank correlations among different factors, Supplementary Material). Finally, five plot-level biotic variables (total basal area, rarefied tree and shrub species richness, tree functional diversity, leaf functional diversity, and deciduousness) as well as seven abiotic variables (soil openness, inclination, elevation, soil pH, maximum air temperature, mean annual relative humidity and minimum annual relative humidity) were used in the final analysis (**Table 1**). Total basal area was defined as the cumulated area of the cross-section of all tree trunks and stems measured at breast height per plot area and in this study basal area was measured in the central 10 m× 10 m area of every plot. Tree and shrub richness was rarified to 150 individuals per 30 m× 30 m. Tree functional diversity was measured for each plot by calculating Rao’s Quadratic Entropy. The index takes the relative abundances of tree species and the pairwise functional differences between tree species into account based on all tree traits. Leaf functional diversity was similar to tree functional diversity but only based on leaf traits. Deciduousness was the proportion of deciduous species in the tree and shrub layer. Most variables describing tree and shrub diversity (rarefied tree richness to 100 and 150 individuals, Shannon diversity, Simpson’s index, species richness, phylogenetic diversity, Shannon evenness) were highly positively correlated (ρ > 0.70, *P* < 0.001); thus, only rarefied richness to 150 individuals was retained. Basal area was highly positively correlated with forest successional stage (ρ = 0.90, *P* < 0.001) and forest age (ρ = 0.91, *P* < 0.001), and negatively correlated with tree and shrub abundance (ρ = -0.76, *P* < 0.001). Thus, only basal area was kept as representative of forest growth. Mean (ρ = -0.86, *P* < 0.001) and minimum (ρ = -0.85, *P* < 0.001) temperature were highly negatively correlated with elevation, and were thus removed from the final analysis. We tested for the effect of space on the WIF community by using different parameters, including geographical coordinates and distances between deadwood samples. The results showed clearly that in our experiment space has no significant effect on WIF community (Supplementary Table S1). This result is consistent with a recent study on the effect of geographical distances in *Fagus sylvatica* logs across three regions of Germany (0.315–626.9 km) (Purahong et al., 2016a). Thus parameters related to space were removed from our analysis.

**Table 1:** Plot-specific biotic and abiotic variables examined in this experiment (SD = standard deviation, Min = minimum, Max = maximum).

Plot factors	Mean	*SD*	Min	Max	Level of measurement
**Biotic**					
Basal area (m^2^)	2.15	1.29	0.22	4.93	Ratio scale
Tree and shrub richness (Rarefy 150)	28.32	6.99	16.35	43.24	Ratio scale
Leaf functional diversity	0.34	0.03	0.27	0.42	Ratio scale
Functional diversity	0.35	0.03	0.29	0.42	Ratio scale
Deciduousness	0.18	0.2	0.02	0.92	Ratio scale
**Abiotic**					
Elevation (m.a.s)	547	168	251	903	Ratio scale
Inclination (slope) (°)	35.19	7.81	20	50	Ratio scale
Soil pH	4.57	0.26	4.12	5.07	interval scale
Openness (%)	8.53	1.45	5.9	13.09	Ratio scale
Maximum temperature (°C)	32.59	1.23	29.89	34.44	Interval scale
Mean Rh (%)	88.33	2.45	84	93.28	Ratio scale
Minimum Rh (%)	22.17	3.58	17.62	31.29	Ratio scale


### Deadwood Experimental Set-up and Sampling

We used freshly cut stem wood of *S. superba* and *P. massoniana* trees that had been harvested in the vicinity of the study area prior to the experiment. For both tree species, the wood came from two sites that are located within a short distance (approximately 5 km radius). In total, 20 trees per species were used for the experiment. Trees that were obviously infected by fungi and xylobionts were excluded from the experiment. The diameter of trees was restricted to 10 ± 2 cm and each sample was sawed to a standard length of 25 ± 1 cm. The mean diameter at breast height across the 27 permanent study plots in 2010 was 17 ± 10 cm for *P. massoniana* and 13 ± 9 cm for *S. superba*. This is larger than the diameter of our samples, however, we were interested in the relative difference in decay rates as well as fungal community composition across our study plots and their relation to tree species diversity and abiotic and biotic factors, rather than in a comprehensive inventory assessment of wood decaying fungi of the region. Bags of two mesh sizes (small = 0.25 mm, large = 7 mm) were used to investigate the impact of insect exclusion on the abundance of WIF. A total of 115 deadwood samples (*P. massoniana*: 58; *S. superba* 57) were placed on the forest floor across all study plots between July 9 and August 2, 2010 (see detailed experimental design in Supplementary Table S2). Wood samples were randomized across plots. Subsamples of dried wood were retained to determine initial wood chemical properties. The samples were retrieved from the forest plots after 2 years. Each sample was carefully removed from the forest floor and placed in a clean plastic bag to avoid loss of debris during transport to the field laboratory, where the samples were processed immediately. All extraneous organic material adhering to the outside of the litterbags was removed and the deadwood samples were weighed to determine mass loss. Of each deadwood sample, two slices (2 cm thick) were sawed, one from the margin and one from the center of the sample. Parts of these subsamples were used to determine the individual water content of each decomposed sample to allow decomposition rate calculation on a dry weight basis. Decomposition rates were calculated as negative exponential function of mass per year for each sample (Olson, 1963). The remainder of each subsample was kept frozen at -20°C and transported on dry ice to Germany for further analysis.

### Wood Chemical Analysis

Initial total C and N contents were determined with an elemental analyzer (Elemental analyzer VarioEL CNS). Total lignin content of undecomposed deadwood was determined following *NREL/NIST* (Sluiter et al., 2010). Wood chemical properties are shown in **Table 2**.

**Table 2:** Wood properties of *P. massoniana* and *S. superba* deadwood (SD = standard deviation, Min = minimum, Max = maximum).

Wood properties	*Pinus massoniana*				*Schima superba*				Level of measurement
			
	Mean	*SD*	Min	Max	Mean	*SD*	Min	Max	
Wood pH (decomposed wood)	4.06	0.33	3.25	4.92	4.22	0.68	3.24	6.59	Interval scale
Mean initial C (%)	49.52	2.70	47.89	58.51	47.99	0.20	47.35	48.26	Ratio scale
Mean initial N (%)	0.04	0.01	0.03	0.06	0.05	0.01	0.04	0.07	Ratio scale
Initial C:N	1252.78	277.94	814.42	1950.17	1000.27	142.32	675.37	1306.75	Ratio scale
Total initial lignin (%)	29.38	1.85	26.35	33.92	27.33	0.80	25.64	29.10	Ratio scale
Decomposition rate (year^-1^)	0.18	0.10	0.01	0.41	0.20	0.07	0.05	0.40	Ratio scale
Remaining mass (%)	64.97	13.91	38.00	91.43	60.82	9.57	39.04	83.55	Ratio scale


### Microbial DNA Extraction and Sequence Library Preparation

Each wood sample was separately homogenized and ground to fine powder with the aid of liquid nitrogen, using a swing mill (Retsch, Haan, Germany). DNA was extracted from 100 mg of each homogenized frozen deadwood sample using a ZR Soil Microbe DNA MiniPrep kit (Zymo Research, Irvine, CA, USA) according to the manufacturer’s protocol. The presence and quantity of genomic DNA was checked using a NanoDrop ND-1000 spectrophotometer (Thermo Fisher Scientific, Dreieich, Germany). DNA extracts were then stored at -20°C for further analysis.

Fungal Amplicon libraries were amplified for pyrosequencing using custom fusion primers. We used the primer pairs ITS1F (5′-CTTGGTCATTTAGAGGAAGTAA-3′, Gardes and Bruns, 1993) and ITS4 (5′-TCCTCCGCTTATTGATATGC-3′, White et al., 1990) to amplify the fungal ITS rRNA region. The custom primers were constructed with the sequencing primers attached at the 5′ end of each fungal specific primer, intercalated with a 10 nt barcode for the ITS4 primer for unidirectional sequencing (Wubet et al., 2012; Lentendu et al., 2014). We thus, targeted the ITS2 region. PCR conditions are described in the Supplementary Material. Amplification products were visualized with a 1.5% agarose gel. The amplified products of the expected size (from 300 bp to 1 kb) from the three positive replicate reactions per sample were then pooled in equimolar amounts and each pool was cleaned using a Qiagen Gel Extraction Kit (Qiagen, Hilden, Germany). This was done to remove short length sequences or primer dimers. Since we removed the short-length sequences, we may remove some few WIF taxa, which have short ITS fragments. The purified DNA was quantified using a fluorescence spectrophotometer (Cary Eclipse, Agilent Technologies, Waldbronn, Germany). An equimolar mixture of each library was subjected to unidirectional pyrosequencing from the ITS4 end of the amplicons, using a 454 Titanium amplicon sequencing kit and a Genome Sequencer FLX 454 System (454 Life Sciences/Roche Applied Science) at the Eurofins Scientific laboratory.

### Bioinformatics Analysis

The raw reads were first demultiplexed and quality trimmed using MOTHUR (Hoppe et al., 2016). Briefly, reads holding one of the expected barcodes with at most one mismatch, a minimum length of 360 nt, a minimum average Phred score of 20, containing homopolymers with a maximum length of 15 nt, and without ambiguous nucleotides were considered for further analyses. The quality-filtered reads were shortened to their first 360 bases and normalized to the smallest read per sample (3,077). Potential chimeras were removed using UCHIME (Edgar et al., 2011) as implemented in MOTHUR. Unique sequences were sorted by decreasing abundances and were clustered into Operational Taxonomic Units (OTUs) using CD-HIT-EST (Fu et al., 2012) at a threshold of 97% pairwise similarity. Fungal ITS OTU representative sequences were first classified against the dynamic version of the UNITE fungal ITS sequence database (version 6, released on 15.01.2014; Kõljalg et al., 2013). The sequences with fungi only identified were further classified against the full version of the UNITE database to improve their taxonomic annotation. *Resinicium* spp. were assigned to order Hymenochaetales and family *Rickenellaceae* according to Mycobank and a molecular phylogeny of the hymenochaetoid clade (Larsson et al., 2006). Rare OTUs (singletons to tripletons), which could potentially originate from artificial sequencing errors (Kunin et al., 2010), were removed from the dataset. Thus we used the abundant OTUs (OTUs with > 3 reads) for further statistical analysis. To assess the role of the omitted rare OTUs, a Mantel test based on a Bray–Curtis distance measure with 999 permutations was applied to both the full matrix and the one excluding the rare OTUs (Hammer et al., 2001; Hoppe et al., 2016; Purahong et al., 2016b). The result indicated that the removal of rare OTUs from the WIF communities had no effect on community composition (*R*_Mantel_ = 0.999, *P* = 0.001). Finally, representative sequences of the fungal OTUs were assigned to functional or ecological groups on the basis of sequence similarity using the default parameters of the GAST algorithm (Huse et al., 2008) against the reference dataset provided by Tedersoo et al. (2014). The raw sequence datasets are available in the European Nucleotide Archive under the study number PRJEB8978^1^. Fungal biom OTU table, OTU representative sequences and the bioinformatic scripts are available at Dryad^2^. The representative sequences and fungal taxonomic table are also available in the Supplementary Material.

### Statistical Analysis

All datasets related to fungal OTU richness were tested for normality and equality of variances using the Jarque–Bera test. To assess the coverage of the sequencing depth, individual rarefaction analysis was performed for each sample using the “diversity” function in PAST program (Hammer et al., 2001; Supplementary Figure S1). In this work, we used observed fungal OTU richness as the measure for fungal diversity. An exploratory NMDS analysis of the plot specific community composition revealed that the mesh size treatments had no effect on the fungal community composition in both tree species (**Table 3**). We thus pooled the fungal OTU data from the fine and coarse mesh treatments for the subsequent WIF OTU richness analyses and will focus on tree species specific differences. The difference in fungal OTU richness between the two deadwood species was compared using a two-sample *t*-test in PAST. Correlations of Fungal OTU richness with biotic and abiotic variables were calculated using non-parametric Spearman’s rank correlation (ρ).

**Table 3:** Goodness-of-fit statistics (*R*^2^) for factors fitted to the three dimensional non-metric multidimensional scaling (3D-NMDS) ordination of fungal community composition.

Factor	All		*Pinus massoniana*		*Schima superba*	
			
	*R*^2^	*P*	*R*^2^	*P*	*R*^2^	*P*
**Wood chemical properties**						
Tree species	0.594	**0.001**	nd	nd	nd	nd
Wood pH	0.371	**0.001**	0.383	**0.001**	0.413	**0.001**
Initial N	0.164	**0.001**	0.040	0.541	0.049	0.437
Initial C: N	0.204	**0.001**	0.026	0.694	0.050	0.430
Total initial lignin	0.213	**0.001**	0.037	0.558	0.026	0.700
**Biotic factor**						
Mesh (insect exclusion)	0.002	0.969	0.016	0.839	0.014	0.860
Basal area	0.069	**0.038**	0.067	0.284	0.044	0.506
Tree and shrub richness (Rarefy 150)	0.001	0.981	0.001	0.999	0.012	0.876
Leaf functional diversity	0.023	0.454	0.081	0.208	0.068	0.282
Functional diversity	0.004	0.948	0.059	0.346	0.053	0.445
Deciduousness	0.016	0.634	0.006	0.961	0.100	0.138
**Abiotic factor**						
Openness	0.019	0.558	0.041	0.518	0.027	0.713
Inclination (slope)	0.119	**0.007**	0.192	**0.012**	0.099	0.143
Elevation	0.090	**0.017**	0.099	0.137	0.134	**0.045**
Soil pH	0.072	**0.048**	0.060	0.361	0.238	**0.002**
Maximum temperature	0.013	0.705	0.146	**0.034**	0.033	0.599
Mean relative humidity	0.052	0.123	0.020	0.786	0.049	0.434
Minimum relative humidity	0.031	0.345	0.096	0.144	0.024	0.754


*Significant *P* values (*P* < 0.05) are indicated in bold; nd, not determined.*

To visualize the WIF community compositions, we used three dimensional non-metric multidimensional scaling (3D-NMDS) analysis based on the Bray–Curtis dissimilarity index calculated in R (R Development Core Team, 2015). The stress values from 3D-NMDS were lower than 0.20 in all cases. To test for significant difference in WIF community composition between the two deadwood species we used non-parametric multivariate analysis of variance (NPMANOVA, 999 permutations) based on Bray–Curtis (relative abundance OTU matrix) and Jaccard (presence-absence OTU matrix) dissimilarity indices. Biotic and abiotic factors (including geographical coordinates and distances between deadwood samples) and chemical properties of the deadwood were fitted to the NMDS ordination plots using the ‘envfit’ function in the vegan package of R, and Goodness-of-fit statistics (*R*^2^) were calculated with *P* values based on 999 permutations (Oksanen, 2013). Deadwood species was incorporated as an explanatory factor to account for all unmeasured tree species specific traits. All significant factors (*P* < 0.05) were plotted in the respective NMDS ordination plots and analyzed by distance-based redundancy analysis (dbRDA), using the Bray–Curtis dissimilarity index with the function capscale of vegan to determine the most influential factors on the fungal community composition (Wu et al., 2013). These procedures were undertaken both using the full dataset (two deadwood species) and separately for each deadwood species. Correlations between (i) total and saprotroph WIF OTU richness and decomposition rates, (ii) relative abundances between *Xylaria* Otu01638 and *Phanerochaete* Otu03458 detected in *S. superba* deadwood and (iii) relative abundances of *Xylaria* Otu01638 and *Phanerochaete* Otu03458 detected in *S. superba* deadwood and wood decomposition rates were calculated using non-parametric Spearman’s rank correlation (ρ).

## Results

### Bioinformatics Analysis

A total of 333,332 quality filtered sequences of fungal ITS were obtained after quality trimming and removal of non-target and chimeric sequences, accounting for 3,077 reads per sample. These were then clustered into 3,507 fungal OTUs including 1,743 singletons, 510 doubletons, and 257 tripletons. Finally, based on the analysis of the impact of removing rare (singletons to tripletons) fungal OTUs by using a Mantel test based on the Bray–Curtis distance measure with 999 permutations (*R*_Mantel_ = 0.999, *P* = 0.001), 997 abundant fungal OTUs were retained for further analysis. In the final dataset, we were able to assign 99.8, 97.5, 90.0, 66.2, and 70.6% of the total fungal OTUs at phylum, class, order, family, and genus levels, respectively.

### WIF OTU Richness: Interplay with Biotic and Abiotic Factors

**FIGURE 1:**
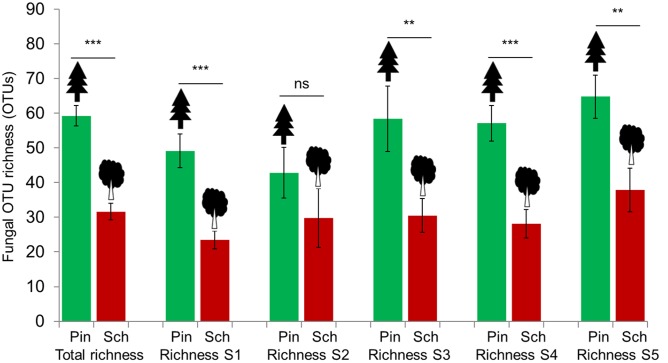
**Comparisons of wood-inhabiting fungal OTU richness between *P. massoniana* (Pin) and *S. superba* (Sch) using total OTUs detected from all stages (S1 – S5) of forest succession as well as each specific stage.** ^∗∗^*P* < 0.01; ^∗∗∗^*P* < 0.001; ns: not significant.

The sample-based rarefaction curves indicated a saturation of fungal richness at the analyzed sequencing depth for most samples, and thus we used the observed OTU richness directly for further analyses (Supplementary Figure S1). The fungal OTU richness differed between the two tree species, but not between mesh treatments within species (*P* > 0.05). The number of fungal OTUs detected in wood of *P. massoniana* was significantly higher than in the wood of *S. superba* (**Figure 1**). WIF OTU richness was usually higher in *P. massoniana* (total = 790; mean = 59.29) than in *S. superba* wood (total = 583; mean = 31.67, *t* = 7.25, *P* < 0.0001) across different forest successional stages, with the exception of the second succession stage (20–40 years, **Figure 1**). Interestingly, we found only a low proportion of fungal OTUs shared between the two deadwood species (376 OTUs, 38%).

When both tree species were considered together, we detected significant positive correlations (*P* < 0.05) between the number of fungal OTUs and wood chemical properties (initial C: N ratio, total lignin content) and plot-level factors (tree and shrub species richness and basal area). Negative correlations were detected between the number of fungal OTUs and initial total N content. Maximum temperature and deciduousness were marginally significantly negatively correlated (*P* < 0.10) with the number of fungal OTUs. However, when the two deadwood species were considered individually, only a few or none of these factors were correlated with fungal richness. Specifically, for *P. massoniana* tree and shrub species richness and basal area were positively correlated with WIF OTU richness, whereas maximum temperature had a negative influence. For *S. superba*, no correlations were found.

We assessed the OTU richness in different fungal phyla and classes. We found that the WIF communities from both deadwood species had slightly higher numbers of OTUs assigned to Ascomycota than Basidiomycota (*P. massoniana*, Ascomycota = 442 OTUs, Basidiomycota = 340 OTUs; *S. superba*, Ascomycota = 305 OTUs, Basidiomycota = 273 OTUs; Supplementary Figures S2, S3). Interestingly, the OTU-rich classes for the two deadwood species were similar: Agaricomycetes (*P. massoniana*: 239 OTUs, represented by the orders Polyporales, Agaricales and Hymenochaetales with 57, 50 and 32 OTUs, respectively; *S. superba*: 218 OTUs, represented by the orders Polyporales, Hymenochaetales and Agaricales with 101, 35 and 31 OTUs, respectively), Sordariomycetes (*P. massoniana*: 141 OTUs, represented by the order Chaetosphaeriales with 52 OTUs; *S. superba*: 145 OTUs represented by the orders Chaetosphaeriales and Hypocreales with 44 and 35 OTUs, respectively) and Leotiomycetes (*P. massoniana*: 141 OTUs, represented by the order Helotiales with 133 OTUs; *S. superba*: 66 OTUs represented by the order Helotiales with 60 OTUs) (Supplementary Figures S2, S3).

### WIF Community Composition

**FIGURE 2:**
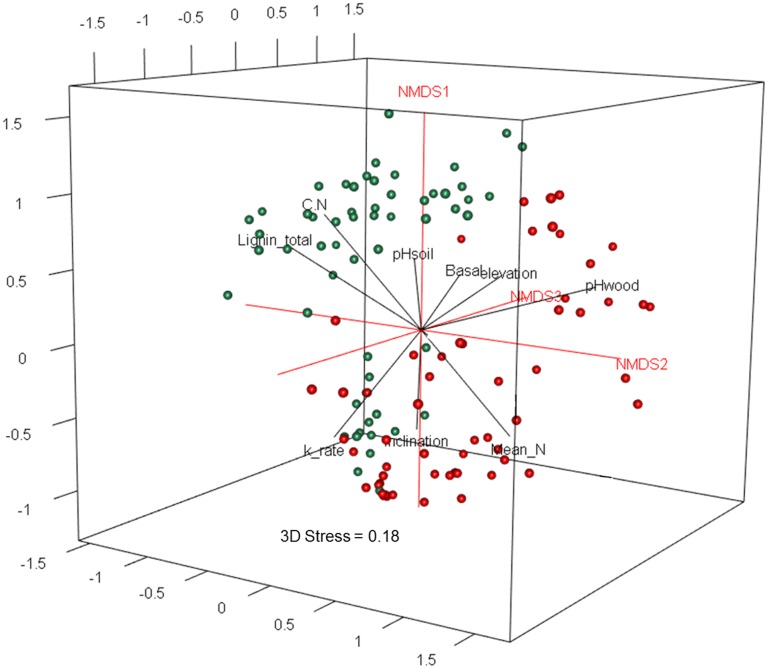
**Three dimensional non-metric multidimensional scaling (3D-NMDS) ordination of wood-inhabiting fungal community composition in *P. massoniana* (green) and *S. superba* (red) deadwood.** The NMDS ordination (stress = 0.18) was fitted to wood chemical properties as well as plot-related biotic and abiotic factors (only significant factors *P* < 0.05 are shown).

**FIGURE 3:**
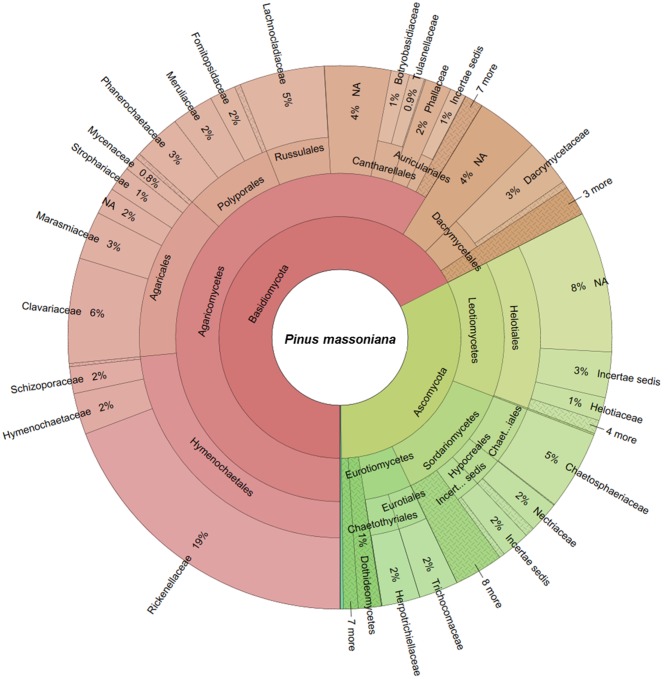
**Wood-inhabiting fungal community composition associated with *P. massoniana* calculated using relative abundance data.** NA = not assigned.

**FIGURE 4:**
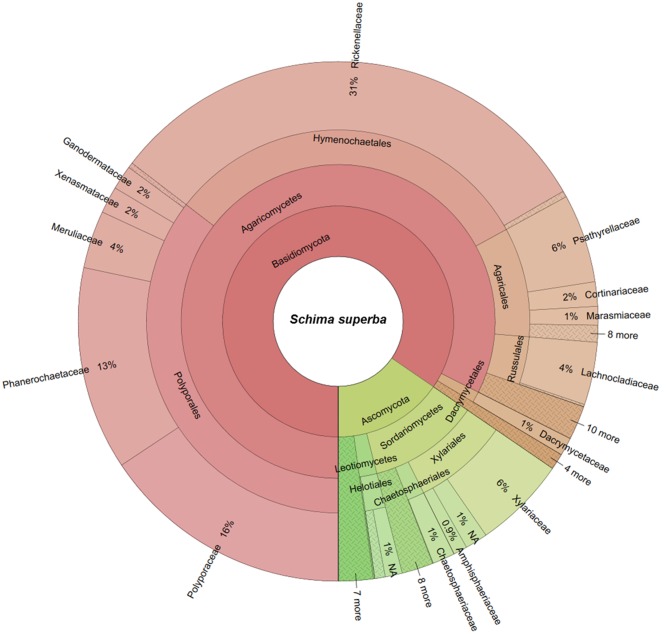
**Wood-inhabiting fungal community composition associated with *S. superba* calculated using relative abundance data.** NA = not assigned.

The two deadwood species had significantly different WIF community compositions at the OTU level (*F* = 5.72, *P* < 0.001; **Figure 2**), while the mesh treatments had no significant effect (**Table 3**). This is reflected in the shift in relative abundance of the two dominant phyla, Ascomycota and Basidiomycota (32.3 and 67.5% of total sequences in *P. massoniana* deadwood and 15 and 85% in *S. superba* deadwood, respectively; **Figures 3**, **4**) but also by changes in community composition within these two phyla. For Ascomycota in *P. massoniana* deadwood, we frequently detected members of Leotiomycetes (13% of total sequences; represented by Helotiales Otu01766), Sordariomycetes (12% of total sequences; represented by *Chaetosphaeria* spp., *Nectria* Otu00714, and *Xylomelasma* Otu10687) and Eurotiomycetes (5% of total sequences; represented by *Penicillium* Otu10681). In *S. superba* deadwood, however, we frequently detected Sordariomycetes (11% of total sequences; represented by *Xylaria* spp.), whilst members of Leotiomycetes and Eurotiomycetes were less frequent or almost absent. The members within Basidiomycota differed greatly between *S.* and *Pinus*, although in both species members of Agaricomycetes (59–82% of total sequences) were highly represented. In *P. massoniana*, we frequently detected *Resinicium* spp. (Hymenochaetales, 19% of total sequences), *Mucronella* spp. (Agaricales, 6% of total sequences) and *Scytinostroma* spp. (Russulales, 5% of total sequences). In *S. superba*, *Resinicium* spp. (Hymenochaetales, 31% of total sequences) were commonly detected along with other Polyporales members (*Tinctoporellus* spp. and *Phanerochaete* spp., with 13 and 10% of total sequences, respectively). All frequently detected fungal OTUs in each deadwood species are shown in the Supplementary Figures S2, S3. Presence–absence data further supported a significant difference in WIF community composition between the two deadwood species (*F* = 5.26, *P* < 0.001).

### WIF Communities: Interplay of Biotic and Abiotic Factors

The fungal community composition was correlated with chemical properties of the two decomposing tree species as well as to biotic and abiotic variables related to the 27 study plots (**Table 3**). These factors included: the chemical properties total initial N content, C: N ratio, total initial lignin content, and pH of the decomposed wood; and the plot-related ecological variables soil pH, tree basal area, elevation and inclination (**Table 3**). Among all variables, the difference of fungal community composition between the two tree species was significantly explained by wood pH, deadwood species, soil pH and tree basal area (*F* = 3.30-6.36, *P* = 0.001–0.005; **Table 4**). The WIF communities identified from each deadwood species were correlated with different abiotic factors (**Table 3**). Only pH of the decomposed wood was correlated with the fungal community compositions in both deadwood species (**Table 3**). The WIF community composition from *P. massoniana* deadwood was related to plot inclination and maximum temperature, whereas the WIF community composition from *S. superba* deadwood was related to plot elevation and soil pH (**Table 3**). Among these factors, inclination was the most important factor significantly correlated with the composition of the *P. massoniana* deadwood fungal community, whereas wood pH and soil pH were the most important factors correlated significantly with the *S. superba* deadwood community (**Table 4**). Interestingly, none of the biotic factors tested had a significant correlation on the WIF communities derived from each deadwood species (**Tables 3**, **4**).

**Table 4:** The most influential factors affecting wood-inhabiting fungal community compositions as determined by distance-based redundancy analysis (db RDA).

Factor	All		*Pinus massoniana*		*Schima superba*	
			
	*F*	*P*	*F*	*P*	*F*	*P*
Tree species	3.642	**0.001**	nd	nd	nd	nd
Wood pH	3.741	**0.004**	ns	ns	2.271	**0.038**
Basal area	3.301	**0.005**	nd	nd	nd	nd
Inclination (slope)	ns	ns	3.876	**0.002**	nd	nd
Soil pH	6.362	**0.001**	nd	nd	2.947	**0.009**
Maximum temperature	nd	nd	ns	ns	nd	nd


*Significant *P* values (*P* < 0.05) are indicated in bold; ns, not significant; nd, not determined.*

### Functional Groups of Wood-Inhabiting Fungi

In general, we identified six functional groups: saprotrophs (647 OTUs, 64.9%), plant pathogens (44 OTUs, 4.4%), ectomycorrhizal fungi (ECM; 21 OTUs, 2.1%), mycoparasites (17 OTUs, 1.7%), lichenized fungi (12 OTUs, 1.2%), animal endosymbionts (1 OTUs, 0.1%), and fungi with unknown functions (255 OTUs, 25.6%; **Table 5**). These fungal functional groups were distributed across different fungal phyla. Animal endosymbionts, lichenized species and plant pathogens were highly represented by Ascomycota (98–100%), while ECM was highly represented by Basidiomycota (90%). Mycoparasites and saprotrophs had similar proportions of Ascomycota (41–50%) and Basidiomycota (50–59%). With the exception of mycoparasites and animal endosymbionts, most functional groups showed a certain deadwood tree species preference, as reflected by the low percentage of shared OTUs between deadwood species (8.3–40.9%). However, the percentage of shared mycoparasitic fungal OTUs was relatively high (58.8%). The only OTU identified as an animal endosymbiont (*Symbiotaphrina* sp.) was found in both deadwood species. Interestingly, we found that deadwood from the ectomycorrhizal host tree (*P. massoniana*) harbored more ECM fungal OTUs (20 OTUs, 95.2%) than the non-ectomycorrhizal host tree (*S. superba*) with only three ECM fungal OTUs.

**Table 5:** Number of OTUs, percent shared and specific OTUs and most abundant detected OTUs of different ecological functional groups of wood-inhabiting fungi (WIF).

Functional group	Number of OTUs	Percent OTUs			Most abundant OTUs (average % relative abundance)		
			
		*Pinus* specific	*Schima* specific	Shared	*Pinus* specific	*Schima* specific	Shared
Animal endosymbiont	1	0	0	100	–	–	*Symbiotaphrina* Otu11126 (0.002)
Ectomycorrhiza	21	85.7	4.8	9.5	Sordariales Otu10857 (0.0501)	Hysterangiales Otu11292 (0.03)	Hysterangiales Otu00521 (0.440)
Lichenized	12	58.3	33.3	8.3	*Sarea* Otu10875 (0.028)	Lecanorales Otu10764 (0.177)	*Sarea* Otu10746 (0.116)
Mycoparasite	17	23.5	17.6	58.8	*Tremella* Otu10745 (0.075)	*Hypocrea* Otu00162 (0.092)	*Cuniculitrema* Otu10698 (0.233)
Plant pathogen	44	45.5	13.6	40.9	*Cosmospora* Otu02936 (0.0201)	*Fusarium* Otu10757 (0.094)	*Pestalotiopsis* Otu02571(0.716)
Saprotroph	647	38.6	23.2	38.2	Cystofilobasidiales Otu10731 (0.243)	*Cystidiodontia* Otu10714 (0.178)	*Resinicium* Otu00870 (25.003)
Unknown	255	45.1	16.9	38.0	Agaricomycetes Otu01452 (0.136)	Dacrymyces Otu10712 (0.697)	*Phanerochaete* Otu03458 (5.482)


### Relationships between Fungal Richness, Community Composition, and Wood Decomposition Rate

**FIGURE 5:**
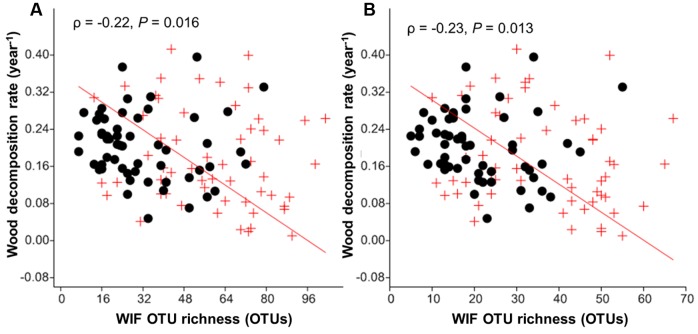
**Correlation between wood decomposition rate and wood-inhabiting fungal (WIF) OTU richness** **(A)** total richness, and **(B)** Saprotroph richness. *P. massoniana* (red crosses) and *S. superba* (black dots) deadwood.

The wood decomposition rates were significantly correlated with WIF community composition in both tree species (*P* = 0.022–0.001; **Table 6**). Furthermore, dbRDA showed that variation in the WIF community composition corresponded significantly to wood decomposition rates when both tree species were considered together (*F* = 3.58, *P* = 0.004) or separately (*F* = 2.73–2.82, *P* = 0.019–0.021; **Table 6**). However, fungal richness was negatively correlated with wood decomposition rates (ρ = -0.22, *P* = 0.016; **Figure 5A**). This result was consistent for the relationship between saprotrophs richness and wood decomposition rates (ρ = -0.23, *P* = 0.013; **Figure 5B**). In general, our results highlight the significance of the deadwood mycobiome and the relationship between fungal richness, community composition and wood decomposition rates in this subtropical forest.

**Table 6:** Correlations between decomposition rates and wood-inhabiting fungal community composition.

Source of fungal community	Goodness-of-fit statistics fitted to 3D-NMDS		Distance-based redundancy analysis	
		
	*R*^2^	*P*	*F*	*P*
All tree species	0.178	**0.001**	3.577	**0.004**
*Pinus massoniana*	0.189	**0.022**	2.820	**0.019**
*Schima superba*	0.335	**0.001**	2.729	**0.021**


*Significant *P* values (*P* < 0.05) are indicated in bold.*

## Discussion

In this study, we combined a controlled deadwood decomposition experiment with a pyrotag sequencing approach in order to investigate wood-inhabiting mycobiome or WIF community compositions of two different wood species and explore their relationships with biotic and abiotic factors in a highly diverse subtropical forest. Keeping possible biases inherent to molecular techniques in mind (Tedersoo et al., 2010; Voříšková and Baldrian, 2013; Lücking et al., 2014), our work provides important and novel insights into the distribution and diversity patterns of WIF in this largely unexplored biome. Besides providing an in-depth picture of the WIF community in this subtropical forest, by comparing our results to results from temperate and boreal forests we also contribute to a better understanding of patterns of WIF communities across different biomes and geographic locations.

### Highly Diverse Wood-inhabiting Fungal Communities

Our study revealed highly diverse wood inhabiting fungal communities with different functional groups in two representative tree species of this subtropical forest. Despite the controlled nature of our experiment with only two tree species, that have been deployed for equal amount of time, and the uniform small size of the samples, it should be made clear that the WIF OTU number in this subtropical biome was high compared to temperate broadleaved and boreal forest biomes (Supplementary Table S3). However, direct comparisons of WIF OTU richness across different studies are difficult and suffer from pitfalls such as the use of different approaches, PCR primers, bioinformatics, and/or deadwood species or genera. Thus we used carefully selected studies for the comparative interpretation of the current results. The species *Resinicium bicolor* (Hymenochaetales), *Fomitopsis pinicola* (Polyporales), and *Heterobasidion* spp. (Russulales) were found in coniferous deadwood(*Picea abies*) across temperate and boreal forests (Kubartová et al., 2012; Ovaskainen et al., 2013; Ottosson et al., 2015; Hoppe et al., 2016) For broadleaved deadwood (from the same genus, i.e., *Fagus* and *Quercus*), there is only information available from temperate broadleaved and mixed forest biomes located in different countries and continents. Interestingly, no fungal OTU has been commonly detected across all locations, thus WIF species may be restricted to certain locations. In the European temperate broadleaved and mixed forest biome, *Trametes versicolor* (Polyporales) and members of Xylariales are frequently detected in *Fagus* and *Quercus* deadwood but are absent or rarely detected in Asian WIF populations, which are mainly dominated by *Neolentinus lepideus* (Agaricales) and *Callistosporium graminicolor* (Agaricales) (van der Wal et al., 2014; Yamashita et al., 2015; Hoppe et al., 2016). Interestingly, in our study *Resinicium* spp. were abundant in both broadleaved and coniferous deadwood. In addition, the members of Xylariales (*Xylaria* spp.) were frequently detected in *S. superba* deadwood, following the pattern of broadleaved deadwood in European temperate broadleaved and mixed forests (van der Wal et al., 2014; Hoppe et al., 2016) but differing from the WIF pattern reported from Asian temperate broadleaf and mixed forest (Yamashita et al., 2015). Comparisons of WIF community derived from conifer and broadleaved deadwood at higher taxonomic levels such as phylum, class and order across different biomes are presented in the section entitled “Pattern of fungal community taxonomic composition across deadwood species and biomes” (Supplementary Material).

Total and *P. massoniana* associated WIF OTU richness increased significantly with tree and shrub species richness, as well as basal area. The significant correlations between WIF OTU richness and tree and shrub species richness may be explained in part by direct plant–fungus interactions as reported for soil and ECM fungi in tropical and subtropical forests (Gao et al., 2013; Peay et al., 2013). Basal area was highly correlated with the number of trees in a plot and forest successional stage. In our study site, the abundance of deadwood increased with successional stage (Wu et al., 2013). According to the species–energy theory, large quantities of deadwood (high available energy) support more species of WIF in tropical forests (Schmit, 2005). We also identified that C: N ratio, total lignin and initial N content of the deadwood were correlated with WIF OTU richness. While our study was not designed to present a mechanistic explanation of how chemical wood properties influence fungal communities in dead wood, these factors include macronutrients that are required for large amounts of fungal growth and reproduction (C and N) and also the main carbon sources of some WIF species (lignin) (Prescott et al., 1999). Furthermore, these factors are most likely to contribute to species specific differences in WIF OTU richness, as *P. massoniana* (higher WIF OTU richness) has higher C: N ratio and total lignin, and lower initial N content than *S. superba* (lower WIF OTU richness).

### Wood-Inhabiting Fungal Communities Responded Differently to Multiple Biotic and Abiotic Factors

The composition of the wood-inhabiting fungal community was significantly correlated to biotic and abiotic factors across both deadwood species. Basal area was the biotic factor that correlated best with the WIF community. WIF were reported to prefer certain successional forest stages, especially the late stage (Junninen et al., 2006). We confirm this result as we found that 78 and 32 WIF OTUs (5.5–9.9%) positively correlated with basal area in *P. massoniana* and *S. superba*, respectively (data not shown). Furthermore, we found that some WIF OTUs [28 and 15 OTUs (2.6–3.5%) in *P. massoniana* and *S. superba*, respectively] negatively correlated with basal area (preferably early forest successional stages). In the same plots, basal area was also found to influence soil and ECM fungal communities significantly (Wu et al., 2013). Insect exclusion did not significantly affect the WIF community in this ecosystem, which indicates that WIF mainly colonize deadwood by other means (soil, water, and wind) rather than by insect vectors (Dighton and White, 2005; Peay et al., 2012). This is confirmed by the fact that the majority of the top 20 relatively most abundant WIF OTUs in both deadwood species were detected in both coarse and fine mesh bags and only one fungal OTU (*Phallus impudicus* Otu01000) from *P. massoniana* was detected exclusively from coarse mesh bags but was absent from the insect exclusion treatment (Supplementary Table S4). *P. impudicus* (common stinkhorn) is known to produce a sticky spore mass with a strong smell to attract flies and other insects (especially Dipterans) (Smith, 1956; Borg-Karlson et al., 1994). In addition, fungal OTU known as insect endosymbiont in the two tree species were identified in both treatments (**Table 5**). Abiotic factors (inclination and elevation) as well as previously known wood-inhabiting fungal correlating factors from temperate and boreal forests (wood chemical factors: N, C:N ratio, total lignin, wood pH, as well as soil pH and deadwood species identity) were found to be significantly correlated with WIF community composition in this subtropical forest (Rajala et al., 2010, 2012; Hoppe et al., 2016). The effects of inclination (slope) and elevation on WIF community are possibly indirect as they might alter other environmental factors. Inclination can induce changes in soil chemical properties (i.e., pH and available phosphorus) and also the plant community composition (Chu et al., 2016). Temperature (air and soil), soil pH, soil moisture, soil chemical properties (e.g., C: N ratio), relative humidity, and plant community were reported to change with elevation (Geml et al., 2014; Looby et al., 2016). Interestingly, WIF communities derived from the two deadwood species correlated with different abiotic factors, while none of the biotic factors correlated with the WIF community pattern within either of the two deadwood species. This finding corroborates results from a recent study in a temperate forest, where two deadwood species with different wood traits (conifer vs. broadleaved; chemical compositions) were found to be associated with different WIF communities. The different WIF community, in turn, correlated with different abiotic factors (Hoppe et al., 2016). Nevertheless, experiments with a wider range of tree species covering a broader spectrum of wood traits are needed to evaluate the general correlations between wood chemical and physical properties and WIF community composition in both temperate and subtropical biomes.

### Diverse Functional Groups Inhabit Subtropical Deadwood

In total we detected six functional groups of fungi in the two tree species and different proportions of fungal phyla within these functional groups. This result is comparable to a recent study on ecological roles of fungi in boreal and hemi-boreal forests (Ottosson et al., 2015). High amounts of detected WIF functional groups indicate the functional importance of deadwood in maintaining and regulation of biodiversity and ecosystem functions in subtropical forests. WIF saprotrophs are the most dominant group as they rely on carbon and other nutrient sources from deadwood (Hoppe et al., 2016). WIF saprotrophs co-occur with insects and lichens in deadwood, which explains why mycoparasites, lichenized fungi, and animal endosymbionts were also detected as part of the WIF community (Floren et al., 2015; Ottosson et al., 2015). Plant fungal pathogens and ECM may switch their lifestyle to act as facultative saprotrophs on deadwood or they use deadwood as shelter waiting for suitable environmental conditions and host tree species (Maharachchikumbura et al., 2014; Lindahl and Tunlid, 2015).

*Symbiotaphrina* (Otu11126) was the only OTU classified as an animal endosymbiont and was detected in very low proportions in both *P. massoniana* and *S. superba*. Members of the genus *Symbiotaphrina* are reportedly obligate gut endosymbionts of a polyphagous coleopteran of the family Anobiidae, a group that includes many species capable of digesting stored-products, like dried plant and woody substrates (Noda and Kodama, 1996; Oppert et al., 2002). It is likely that we detected *Symbiotaphrina* from the fragments of eggs or egg cases of hatched larvae (Noda and Kodama, 1996). In this study, 12 ascomycota fungal OTUs were classified as lichenized. Of these OTUs the genus *Sarea* was predominantly found in *P. massoniana* while OTUs from the order Lecanorales were only found in *S. superba*. The detection of the DNA of lichenized fungi may originate from vegetative propagules or dispersed thallus fragments (Ottosson et al., 2015). A dimorphic fungus *Cuniculitrema* Otu10698 was the most frequently detected mycoparasite in both deadwood species. Bark beetles have been found to be the vector for this mycoparasite (Kirschner et al., 2001). *Pestalotiopsis* Otu02571 was among the most frequently detected plant pathogens in both tree species. Members of the genus *Pestalotiopsis* are common phytopathogens and endophytes. However, because of their ability to switch between nutritional modes, many *Pestalotiopsis* species can persist as saprobes in dead leaves and wood (Maharachchikumbura et al., 2014). The frequent detection rates of *Pestalotiopsis* Otu02571 as well as other plant fungal pathogens, which may have the ability to switch their life style to that of a saprotroph, could suggest that deadwood might serve as an inoculum source of plant pathogenic fungi (Maharachchikumbura et al., 2014).

Among the ECM OTUs, Hysterangiales Otu00521 had the highest average relative abundances in both deadwood species. However, we only detected Hysterangiales Otu00521 in four samples (two samples for each deadwood species) but with extremely high relative abundance (15–34%). Members of the Hysterangiales are truffle-like and are considered obligate ectomycorrhizal fungi (Hosaka et al., 2008). Thus, it is surprising to detect Hysterangiales OTUs in deadwood. Thelephorales was the richest order of fungi identified as ECM (10 OTUs in total) and most of these OTUs (9/10 OTUs) were detected only in *P. massoniana*. Although we detected many ECM OTUs in deadwood, their role in deadwood decomposition is still unclear (Rajala et al., 2012). The largest and most important functional group detected in this study was the saprotrophs (65% of all detected OTUs) which are crucial for deadwood decomposition. *Resinicium* Otu00870 was the dominant OTU in both deadwood species. *Resinicium* spp. are white rot fungi that are highly abundant in various decay stages of coniferous deadwood in temperate as well as boreal forests (Kubartová et al., 2012; Ottosson et al., 2015; Hoppe et al., 2016). Although five out of six functional groups detected in this study may not play direct role in deadwood decomposition, different fungal functional groups may have influenced deadwood decomposition rates by mediating the interactions between plants, arthropods and fungal saprotrophs. Thus, functional assignments of WIF fungal communities are of crucial importance to increase our understanding of the complex deadwood decomposition processes (Ottosson et al., 2015).

### Tree Species Preference across Different Fungal Functional Groups

In contrast to our initial hypothesis assuming low specialization of fungi due to the high tree diversity in subtropical forests with a wider spatial distribution of tree species, our results showed that WIF in subtropical forests potentially exhibit tree species preference. Our results on tree species preference were consistent across different wood-inhabiting functional groups, except for mycoparasites and animal endosymbionts. It is possible that the opportunities for WIF to find suitable tree species are increasing due to the high abundance of *P. massoniana* and *S. superba* in this region. Fruit body survey-based studies have suggested that WIF, especially polypore fungi in tropical forests, constitute a reservoir of rare species, while more common WIF species are non-specialists (or exhibit no tree species preference) due to tree species rarity (Gilbert and Sousa, 2002). Our results generally confirm this finding for subtropical forests, however, this was not the case for WIF polypores. WIF polypores represent a great proportion of many rare species, but in our study more commonly detected WIF polypore species account for similar proportions of non-specialists and specialists (Gilbert and Sousa, 2002). Our results can only provide a hint about tree species preferences in WIF, since we only considered two species as substrates. Further studies with a larger number of deadwood species are needed to reach conclusions about tree species specificity or preference of WIF in subtropical forests.

Interestingly, we found that the mycorrhizal status of a tree species strongly correlated with richness of ectomycorrhizal fungi in deadwood. *P. massoniana* (an ECM tree species) harbored a wide variety of ECM OTUs, while *S. superba* (a non-ECM tree species) only contained a very small number of ECM OTUs. There is still no explanation of how dead trees can mediate the colonization of ECM that generally forms a symbiotic relationship with living plants. In dead plant material, ECM fungi potentially act as facultative saprotrophs compared to obligate mycorrhizae in living plants (Lindahl and Tunlid, 2015). The relationship between plant and fungal richness has been reported to be stronger for symbiotic fungi than for saprotrophs (Peay et al., 2013), and thus we expected a weak effect of tree species identity on deadwood ECM richness. However, in this study we found the opposite relationship where the deadwood ECM fungi may exhibit a preference for an ECM tree species. Nevertheless, more similar studies using more tree species are needed to confirm this finding.

### Links between Fungal Diversity, Community Composition, and Wood Decomposition Rate

**FIGURE 6:**
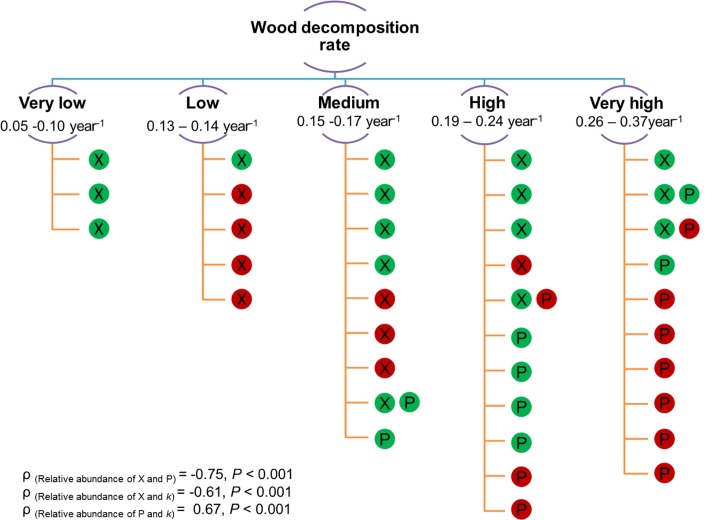
**Presence of *Xylaria* Otu01638 (X) and *Phanerochaete* Otu03458 (P) detected in 38 *S. superba* deadwood samples arrange according to increased wood decomposition rate (*k* rate; from very low to very high by means of *k*-means clustering).** Filled color in each circle indicates the relative abundance of each fungal OTU (green = below 10% and red = above 10%). Spearman’s rank correlations (*n* = 38) between (i) relative abundances of *Xylaria* Otu01638 and *Phanerochaete* Otu03458, (ii) relative abundances of *Xylaria* Otu01638 and *k* rate and (iii) relative abundances of *Phanerochaete* Otu03458 and *k* rate are shown.

In our study, we found significant correlations of fungal richness (total and saprotroph) and community composition with decomposition rate (**Figure 5** and **Table 6**). We expected a negative impact of fungal diversity on the wood decomposition rate due to the very high diversity of different functional groups WIF, which was supported by our results. WIF may invest more energy and resources in competition than in producing wood-degrading enzymes (Fukami et al., 2010; Dickie et al., 2012). Furthermore, we found that interspecific interactions among WIF and competition scenarios may play important roles in deadwood decomposition in a sub-tropical forest, which is consistent with observations from temperate forests (Hoppe et al., 2016). In particular, the significant negative correlations between dominant OTUs support the hypothesis of competition, which in turn influenced wood decomposition rates (Supplementary Table S5). For example, in *S. superba* deadwood, relative abundances of *Xylaria* Otu01638 were negatively correlated with relative abundances of *Phanerochaete* Otu03458 and Sordariales Otu04020 as well as with decomposition rates (**Figure 6** and Supplementary Tables S5, S6). Conversely, there were positive correlations between the relative abundance of *Phanerochaete* Otu03458 and Sordariales Otu04020 with wood decomposition rates. The positive correlation between the relative abundance of *Phanerochaete* Otu03458 and wood decomposition rates was not surprising as *Phanerochaete* spp. comprise many of the white rot fungi (e.g., *Phanerochaete chrysosporium*) which produce various lignin oxidative enzymes (e.g., general peroxidases, lignin peroxidases, manganese peroxidases) as well as hemicellulose and cellulose hydrolytic enzymes (e.g., xylanase, cellulases) (Martinez et al., 2004; Singh and Chen, 2008). The negative correlation between the relative abundance of *Xylaria* spp. and the decomposition rate is consistent with previous observations for *F. sylvatica* logs in temperate forests (Hoppe et al., 2016). This could be related to the ability of *Xylaria* spp. to impede deadwood colonization by secondary saprotrophic basidiomycetes (in this case *Phanerochaete* Otu03458) (Fukasawa et al., 2009; Purahong and Hyde, 2010; Hoppe et al., 2016).

## Conclusion

Deadwood in subtropical forests harbors a high diversity of wood-inhabiting mycobiome with different ecological functions. Previous studies in temperate and boreal forests have shown that WIF community composition is correlated with various abiotic factors, especially wood physical and chemical properties. Our current study in a subtropical forest confirms this significant correlation to wood chemical properties (especially wood pH), which may imply that these correlations are consistent across different biomes. Furthermore, our study revealed new biotic (tree basal area as representative of forest growth and successional stages) and abiotic (inclination and elevation) factors that correlated significantly with WIF community composition. We also identified a unique response of fungal taxonomic groups never reported from other biomes, including *Resinicium* spp. that were most commonly detected in both broadleaved and coniferous deadwood. Our results highlight the relationships between fungal richness/community composition and deadwood decomposition rates in subtropical forest ecosystems. We conclude that the pattern of WIF OTU richness and community composition are controlled by multiple interacting biotic and abiotic factors. Further studies of WIF communities in different biomes are needed to identify their global pattern and the factors shaping their local and global distribution.

## Author Contributions

KAP, CW, FB, TW, HB, GL, and WP conceived and designed the experiments. KAP performed the field experiments. WP and RS performed the laboratory works. WP, TW, and KAP wrote the manuscript. TW, GL, and WP analyzed and interpreted the results; TW, FB, and CW obtained funding. All authors contributed to revisions and gave approval for submission.

## Conflict of Interest Statement

The authors declare that the research was conducted in the absence of any commercial or financial relationships that could be construed as a potential conflict of interest.

## Footnotes

1 http://www.ebi.ac.uk/ena/data/view/PRJEB8978

2 doi: 10.5061/dryad.54qr4
