# Supplementary material for: Characterization of Unexplored Deadwood Mycobiome in Highly Diverse Subtropical Forests Using Culture-independent Molecular Technique
Source: Front Microbiol. 2017 Apr 19;8:574. doi: 10.3389/fmicb.2017.00574 (PMC5395659; doi:10.3389/fmicb.2017.00574)
Supplement: Supplementary file 1 [file Presentation_1.PDF]

Supplementary Material

Characterization of unexplored deadwood mycobiome in highly diverse subtropical forests

Witoon Purahong, Katherina A. Pietsch, Guillaume Lentendu, Ricardo Schöps, Helge Bruelheide,

Christian Wirth, François Buscot, Tesfaye Wubet

Explanations of biotic and abiotic factors

1) Basal area: the cumulated area of the cross-section of all tree trunks and stems measured at breast height per plot area, and in this study basal area was measured in the central 10 × 10 m area of every plot.
Unit: m^2

2) Tree and shrub richness (Rarefy 150): Estimate of species number by randomly drawing 150 individuals (rarefaction). Rarefaction curves show the increase in species number with an increase of sampled individuals.
Unit: count

3) Leaf functional diversity: Leaf functional diversity was measured for each CSP by calculating Rao's Quadratic Entropy. The index takes the relative abundances of species and the pairwise functional differences between species into account based on leaf traits only.

4) Functional diversity: Functional diversity all tree traits. The Functional Diversity (FD) was measured for each CSP by calculating Rao's Quadratic Entropy. The index takes the relative abundances of species and the pairwise functional differences between species into account based on all tree traits.

5) Openness: Soil cover. Estimated in the central plot of the CSP.; Datagroup description: Estimated cover when looking from above. Cover can be estimated in percent.

Unit: percent

6) Inclination: Calculated plot mean slope inclination from geomorphological maps
Unit: degree

7) Elevation: Elevation of the sampled plot, measured with geko201 GPS; Elevation;; ; GIS, Hypsometer, Interpolation from map (derived from datagroup); Instrumentation: GIS, Hypsometer, Interpolation from map (derived from data group).
Unit: m asl

Plot description

Study site characteristic	Description	References
Location	The Gutianshan National Nature Reserve , Zhejiang Province, China	Bruelheide et al., 2011
Plot establishment year	May and July 2008	Bruelheide et al., 2011
Number	27	Bruelheide et al., 2011
Forest classification	Subtropical mixed broad-leaved forest (moist broadleaf forest (<500 m a.s.l.) = 9 plots and montane moist broadleaf forest (>500 m a.s.l.) = 18 plots)	Bruelheide et al., 2011; Schuldt et al., 2015
Size	30m x 30m	Bruelheide et al., 2011
Minimum distance between plots	200 m	Staab et al., 2014
Tree species richness	147 tree species , ranging from 25 to 69 tree species per plot	Staab et al., 2014
Dominant tree species	*Castanopsis eyrei* (Fagaceae), *Schima superba* (Thecaceae), *Pinus massoniana* (Pinaceae), *Daphniphyllum oldhamii* (Daphniphyllaceae)	Bruelheide et al., 2011; Staab et al., 2014
Soil characteristics	Percent clay = 18 - 25% , percent sand = 31 - 56%, percent organic carbon = 4 - 9% , C: N ratio = 16 -22, pH (KCl) = 3.6 - 4	Wu et al., 2013
Site description	The study plots were established in the Gutianshan National Nature Reserve using a stratified sampling design to capture the range of woody plant species richness and successional age (from <20 to >80 years since the last logging events) typically encountered in the reserve. Areas that were inaccessible or had steep slopes>50 ° were excluded. The study plots were randomly spread across the accessible parts of the reserve.	Bruelheide et al., 2011; Staab et al., 2014; Schuldt et al., 2015

PCR conditions

Samples were amplified for pyrosequencing using a forward and reverse fusion primer. We used fusion primers designed with pyrosequencing primer B, a barcode and the fungal specific primer ITS1-F (Gardes and Bruns 1993) as a forward primer and pyrosequencing primer A and the universal eukaryotic primer ITS4 (White et al. 1990) as a reverse primer to amplify the fungal nuclear ribosomal internal transcribed spacer (nrITS) rDNA. We used a set of 10 nt MID-barcodes provided by Roche Applied Science (Mannheim, Germany). Each composite DNA extract for the amplicon libraries was amplified separately by PCR in triplicate 50µl reaction mixtures containing25µl 2x GoTaq Green Mastermix (Promega, Madison, WI,USA), 25µM of each primer and approximately 20 ng template DNA. Amplification was performed using a touchdown PCR program with denaturation at 95 °C for 5 min followed by10 cyclesofdenaturationat94°C for 30 s,annealing at60–50 °C for 45 s (−1 °C per cycle), and extension at 72 °C for 2 min, followed by 30 cycles of 94 °C for 30 s, 50 °C for 45 s and 72 °C for 2 min, with a final 10 min extension step at 72 °C (Lentendu et al. 2014).

Pattern of fungal community taxonomic composition across deadwood species and biomes

Comparisons of WIF community derived from conifer and broadleaved deadwood at higher taxonomic levels such as phylum, class and order across different biomes (Hoppe et al., 2016; Kubartová et al., 2012; Ottosson et al., 2015; Ovaskainen et al., 2013; van der Wal et al., 2014; Yamashita et al., 2015) revealed some interesting colonization and distribution patterns. (i) Ascomycota and Basidiomycota are the most frequently detected phyla. When abundance data are considered Basidiomycota are much more frequently detected than Ascomycota (except for stump deadwood) but when presence/absence data are considered Ascomycota are highly detected in both conifer and broadleaved deadwood in many study locations (Table 7). (ii) Agaricomycetes (Basidiomycota) are among the most frequently detected classes in all deadwood types, biomes and study sites, followed by Leotiomycetes (Ascomycota) which is also highly detected in all biomes but not for all deadwood types and locations. (iii) Agaricales and Polyporales (Basidiomycota) were highly detected in all deadwood types in almost all locations. For Ascomycota, the Helotiales were frequently detected in almost all deadwood types and biomes (except broadleaved deadwood in subtropical forest where Xylariales becomes the highly detected taxa) and (iv) Hymenochaetales (mainly contributes by *Rickenellaceae*, *Resinicium* spp.) is listed among the most frequently detected orders in coniferous deadwood in all biomes and locations but only highly detected in broadleaved deadwood in subtropical forest (Table 7). The distinct pattern of WIF community in subtropical forest compares with other biomes supports that fungal species do not typically have cosmopolitan distributions and the fungal community assembly are strongly affected by biogeographical and environmental factors (i.e. climate, isolation, latitude, temperature, and precipitation). A recent work investigating >52, 000 fungal OTUs across different continents demonstrate that the majority (~ 60 - 80%) of all detected fungal species were unique (endemic) to one continent and dispersal limitation may play important role in maintaining this regional endemism that could impact on local community assembly (Kõljalg et al., 2013). This could explain why certain fungal taxa exist in subtropical forest instead of temperate forest or boreal forest. In addition the result from our study suggests that even though the same WIF taxa (e.g. *Resinicium* spp.) could be presented at all biomes; climate, local environment and tree species could determine their abundances (see table below).

Summary of higher taxonomic pattern of wood-inhabiting fungal community based on high throughput sequencing across different locations/biomes
(Abund = abundance data, Pre-Abs = presence/absence data, A = Ascomycota, B = Basidiomycota, n.a. = not available)

Biomes	Tree species (type)	Sample type	N	Frequently detected phyla		Frequently detected classes (Abund)	Frequently detected orders (Abund)	References
				Abund	Pre-Abs			
Temperate broadleaf and mixed forest (Germany)	*Fagus sylvatica* (broadleaved)	Large logs (various decomposition stages)	24	B > A	A > B	Agaricomycetes (B), Sordariomycetes (A), Leotiomycetes (A)	Agaricales (B), Polyporales (B), Xylariales (A), Russulales (B), Helotiales (A)	Hoppe et al., 2015
Temperate broadleaf and mixed forest (Japan)	*Fagus* spp. (broadleaved)	Trunks and snags(various decomposition stages)	33	B >> A	B >> A	Agaricomycetes (B)	Polyporales (B), Agaricales (B), Phallales (B), Amylocorticiales (B)	Yamashita et al., 2015
Temperate broadleaf and mixed forest (The Netherlands)	*Quercus robur* (broadleaved)	Tree stumps 0, 2 and 5 years after cutting, only sap wood	42	A > B	A > B	Agaricomycetes (B), Leotiomycetes (A)	Helotiales (A), Polyporales (B), Agaricales (B)	van der Wal et al., 2015
Temperate broadleaf and mixed forest (Japan)	*Quercus* spp. (broadleaved)	Trunks and snags (various decomposition stages)	15	B >> A	B >> A	Agaricomycetes (B), Leotiomycetes (A)	Russulales (B), Polyporales (B), Agaricales (B), Helotiales (A)	Yamashita et al., 2015
Subtropical forest (China)	*Schima superba* (broadleaved)	Cut stems (two years decomposition)	57	B >> A	A > B	Agaricomycetes (B), Sordariomycetes (A)	Polyporales (B), Hymenochaetales (B), Agaricales (B), Xylariales (A), Russulales (B)	This study
Hemi-boreal forest (Sweden)	*Picea abies* (coniferous)	Large logs >10 years after cutting	12	B >> A	A > B	Agaricomycetes (B), Leotiomycetes (A)	Polyporales (B), Hymenochaetales (B), Russulales (B), Helotiales (A)	Kubartov´a et al., 2012
Boreal forest (Sweden)	*Picea abies* (coniferous)	Large downed logs, decompose >10 years	26	B > A	A > B	Agaricomycetes (B), Leotiomycetes (A) Eurotiomycetes (A)	Hymenochaetales (B), Helotiales (A), Agaricales (B), Polyporales (B), Russulales (B), Chaetothyriales (A)	Kubartov´a et al., 2012; Ottosson et al., 2015
Boreal forest (Finland)	*Picea abies* (coniferous)	Large logs (various decomposition stages)	100	B >> A	n.a.	Agaricomycetes (B)	Polyporales (B), Russulales (B), Hymenochaetales (B)	Ovaskainen et al., 2013
Temperate broadleaf and mixed forest (Germany)	*Picea abies* (coniferous)	Large logs (various decomposition stages)	24	B >> A	B > A	Agaricomycetes (B), Leotiomycetes (A)	Hymenochaetales (B), Russulales (B), Agaricales (B), Polyporales (B), Hymenochaetales (B), Helotiales (A)	Hoppe et al., 2015
Subtropical forest (China)	*Pinus massoniana* (coniferous)	Cut stem (two years decomposition)	58	B >> A	A > B	Agaricomycetes (B), Leotiomycetes (A), Sordariomycetes (A)	Hymenochaetales (B), Agaricales (B), Helotiales (A), Polyporales (B), Russulales (B), Chaetosphaeriales (A)	This study

Table S1. Effect of space on wood-inhabiting fungal community composition. Geographical coordinates and distances between deadwood samples of the deadwood were fitted to the NMDS ordination plots using the 'envfit' function in the vegan package of R, and Goodness-of-fit statistics (R^2) were calculated with *P* values based on 999 permutations. We also analyzed the effect of these parameters by distance-based redundancy analysis (dbRDA), using the Bray–Curtis dissimilarity index with the function capscale of vegan.

**Factor**	**Overall wood-inhabiting fungal community**			
	R^2	***P***	***F***	***P***
Geographical coordinates (˚N)	0.046	0.174	0.9937	0.460
Geographical coordinates (˚E)	0.026	0.388	1.3998	0.115
Distances between deadwood (Km)	0.009	0.787	1.3979	0.210

Table S2. Experimental design (Stage: successional stage; C: coarse mesh size; F: fine mesh size).

**Deadwood species**	**Number of deadwood**					
	**Total**	**Stage 1 (C/F)**	**Stage 2 (C/F)**	**Stage 3 (C/F)**	**Stage 4 (C/F)**	**Stage 5 (C/F)**
Pinus massoniana	58	12 (6/6)	8 (4/4)	10 (5/5)	14 (7/7)	14 (7/7)
Schima superba	57	12 (6/6)	8 (4/4)	10 (5/5)	14 (7/7)	14 (7/6)

TableS3. Summary of studies on wood-inhabiting fungal community using high throughput sequencing across different locations/biomes

**Biomes**	**Location**	**Tree species**	**Method**	**Sample type**	**Sample size**	**Number of reads**	**Total OTUs (rare OTU removal)**	**Number of OTUs per sample (mean)**	**Frequently detected taxa**	**Factors correlated with WIF community structure**	**References**
Temperate broadleaf and mixed forest	Southwestern Germany	*Fagus sylvatica*	454 pyrosequencing	Large log with various decomposition stages (Length 2 to 24 m , Diameter >7cm)	24	1652	506 (singletons to tripletons)	17 to 102 (58.29)	*Annulohypoxylon cohaerens* (Xylariales), *Hypoxylon rubiginosum* (Xylariales), *Xylaria hypoxylon* (Xylariales), *Trametes versicolor* (Polyporales), *Armillaria* spp. (Agaricales), *Neobulgaria pura* (Helotiales), *Fomes fomentarius* (Polyporales), *Phlebia livida* (Polyporales), *Mycena* spp. (Agaricales), *Megacollybia* spp. (Agaricales)	Decay class, remaining mass, volume, density, relative wood moisture, C (g/cm3), N (g/cm3)	Hoppe et al., 2015
Temperate broadleaf and mixed forest	Southwestern Germany	*Picea abies*	454 pyrosequencing	Large log with various decomposition stages (Length 2 to 25 m , Diameter >7cm)	24	1757	504 (singletons to tripletons)	28 to 102 (65.41)	*Resinicium bicolor* (Incertae sedis), *Amylostereum* spp. (Russulales), *Heterobasidion* sp. (Russulales), *Armillaria* spp. (Agaricales), *Fomitopsis pinicola* (Polyporales), *Mycena* spp. (Agaricales), *Hyphodontia* spp. (Hymenochaetales)	Decay class, remaining mass, density, relative wood moisture, total lignin, pH, C, Mg	Hoppe et al., 2015
Temperate broadleaf and mixed forest	Ogawa Forest Reserve, Pacific Coast of Japan	*Fagus* spp.	Illumina	Trunks and snags with various decomposition stages (length ≥1 m, DBH≥ 11 cm)	33	1603 to 89661	120 (OTUs < 100 reads)	18 to 48	*Phallus hadriani* (Agaricales), *Neolentinus lepideus* (Agaricales), *Callistosporium graminicolor* (Agaricales), *Poria cocos* (Agaricales), and *Ceraceomyces borealis* (Agaricales)	time since death	Yamashita et al., 2015
Temperate broadleaf and mixed forest	Ogawa Forest Reserve, Pacific Coast of Japan	*Quercus* spp.	Illumina	Trunks and snags with various decomposition stages (length ≥1 m, DBH≥ 11 cm)	15	5346 to 40216	112 (OTUs < 100 reads)	20 to 44	*Hericium erinaceum* (Agaricales), Basidiomycete INF1-B (Agaricales), *Neolentinus lepideus* (Agaricales), *Callistosporium graminicolor* (Agaricales), *Artomyces pyxidatus* (Agaricales), and *Neobulgaria premonophila* (Leotiales)	time since death, DBH	Yamashita et al., 2015

Biomes	Location	Tree species	Method	Sample type	Sample size	Number of reads	Total OTUs (rare OTU removal)	Number of OTUs per sample (mean)	Frequently detected taxa	Factors correlated with WIF community structure	References
Temperate broadleaf and mixed forest	The Netherlands	*Quercus robur*	454 pyrosequencing	Tree stumps 2 and 5 years after cutting (length 27 - 50 cm, DBH≥ 15 cm), only sap wood	42	mean 10198 (359 - 17571)	447 (n.a.)	~3 to 100	*Mollisia* sp. (Helotiales), *Trametes versicolor* (Polyporales) , *Panellus stipticus* (Agaricales),	n.d.	van der Wal et al., 2015
Hemi-boreal forest	Baltic coast island in central Sweden	*Picea abies*	454 pyrosequencing	Large log >10 years after cutting (Length > 3m , Diameter > 44cm)	12	mean 469 (varied)	973 (singletons)	~25 to 34	*Fomitopsis pinicola* (Polyporales), *Resinicium bicolor* (Incertae sedis), *Heterobasidion parviporum* (Russulales), *Hyphodontia pallidula* (Hymenochaetales), *Kneiffiella alutacea* (Hymenochaetales), *Phialophora* sp. (Chaetothyriales)	Position among Logs, positions within log (out–in, top-down, base-top)	Kubartov´a et al., 2012; Ottosson et al., 2015
Boreal forest	Northern Sweden	*Picea abies*	454 pyrosequencing	Large downed log >10 years (Diameter > 10cm)	26	mean 785 (varied)	1406 (singletons)	~40 to 50	*Phellinus nigrolimitatus* (Hymenochaetales), *Hyphodontia pallidula* (Hymenochaetales), *Leptodontidium elatius* (Helotiales), *Conferticium ochraceum* (Russulales), *Phialophora* sp. (Chaetothyriales), *Mycena purpureofusca* (Agaricales), *Phellinus* spp. (Hymenochaetales), *Antrodia* spp. (Polyporales),	position among Logs, positions within log (base–top), decay stage	Kubartov´a et al., 2012; Ottosson et al., 2015
Boreal forest	Southern Finland	*Picea abies*	454 pyrosequencing	Large log with various decomposition stages (Length > 15 m , Diameter 20 to 42 cm)	100	mean 3192 (n.d.)	198 (singletons to 4tons)	3 - 40 (16)	*Fomitopsis pinicola* (Polyporales), *Heterobasidion* sp. (Russulales), *Phellinus* spp. (Hymenochaetales), Antrodia (Polyporales), *Resinicium* spp. (Incertae sedis)	n.d.	Ovaskainen et al., 2013

Table S4. Identification of top 20 relatively most abundant WIF OTUs in both deadwood species according to UNITE and NCBI. Abund. = abundance; C = Samples from coarse bags; F = Samples from fine bags (insect exclusion); n.a. = not available.

Ranking	*Pinus massoniana*							
	**OTU (UNITE)**	**Best identified hit (NCBI)**	**Accession number**	**E-value**	**Similarity (%)**	**Mean abund. C**	**Mean abund. F**	**Mean abund.**
1	*Resinicium*Otu 00870	*Resinicium friabile*	KJ831948.1	0	99	14.9	23.3	19.1
2	HelotialesOtu 01766	Helotiales	EU715657.1	0	99	8.47	4.76	6.62
3	*Mucronella*Otu 10332	*Mucronella* sp.	EU770252.1	8E-151	94	4.78	7.22	6
4	*Scytinostroma*Otu 01080	*Scytinostroma* sp.	AB470241.1	0	99	3.03	6.38	4.71
5	*Tubulicrinis*Otu 10683	Basidiomycota	HM240148.1	2E-172	98	2.65	2.19	2.42
6	*Penicillium*Otu 10681	*Penicillium lignorum*	NR_138299.1	6E-127	90	2.37	2.05	2.21
7	AtractiellalesOtu 10692	Atractiellales	GU079602.1	4E-124	89	0.78	3.58	2.18
8	*Phlebia*Otu 02299	*Phlebia* sp.	HE664145.1	0	100	2.28	1.37	1.83
9	*Phanerochaete*Otu 03458	*Phanerochaetaceae*	HM595569.1	0	99	1.69	1.83	1.76
10	*Calocera*Otu 10690	*Calocera cornea*	AB841070.1	0	99	3.41	0.07	1.74
11	AgaricalesOtu 00019	Basidiomycota	KJ654522.1	2E-167	98	0	3.19	1.6
12	*Phallus*Otu 01000	*Phallus indusiatus*	AF324173.2	3E-180	99	3.12	0	1.56
13	*Hyphodontia*Otu 10680	*Hyphodontia palmae*	DQ340333.1	0	99	3.01	0	1.51
14	*Nectria*Otu 00714	*Nectria mariannaeae*	GU586835.1	0	100	2.11	0.89	1.5
15	*Chaetosphaeria*Otu 09386	*Chaetosphaeria bombycina*	NR_119667.1	4E-134	91	1.29	1.58	1.44
16	*Peniophorella*Otu 10699	*Peniophorella praetermissa*	JQ358804.1	3E-174	98	0.68	2.15	1.42
17	***Postia*Otu 05040**	**Antrodia sp.**	JX507729.1	0	100	0	2.75	1.38
18	*Xylomelasma*Otu 10687	*Xylomelasma* sp.	FR837913.1	0	99	0.74	1.88	1.31
19	*Xenopolyscytalum*Otu 00820	*Xenopolyscytalum* sp.	HE603989.1	0	99	1.41	0.81	1.11
20	*Chaetosphaeria*Otu 10709	*Chaetosphaeria bombycina*	NR_119667.1	6E-137	92	0.62	1.51	1.07

Ranking	*Schima superba*							
	**OTU (UNITE)**	**Best identified hit (NCBI)**	**Accession number**	**E-value**	**Similarity (%)**	**Mean abund. C**	**Mean abund. F**	**Mean abund.**
1	*Resinicium*Otu 00870	*Resinicium friabile*	KJ831948.1	0	99	31.5	30.5	31
2	*Tinctoporellus*Otu 02811	*Tinctoporellus epimiltinus*	KP965918.1	0	99	15.5	8.15	11.83
3	*Phanerochaete*Otu 03458	*Phanerochaetaceae*	HM595569.1	0	99	10.7	7.75	9.23
4	*Psathyrella*Otu 00072	*Psathyrella badhyzensis*	KC992883.1	0	99	4.16	5.24	4.7
5	*Scytinostroma*Otu 01080	*Scytinostroma* sp.	AB470241.1	0	99	3.23	4.56	3.9
6	*Xylaria*Otu 01638	*Xylaria* sp.	KT291429.1	0	100	2.38	4.83	3.61
7	*Phlebiopsis*Otu 10679	*Phlebiopsis* sp.	LC013376.1	0	100	0	5.59	2.8
8	*Trametes*Otu 09923	Trametes versicolor	AB733646.1	0	100	1.83	2.71	2.27
9	*Phlebia*Otu 02299	*Phlebia* sp.	HE664145.1	0	100	4.14	0.04	2.09
10	*Xenasmatella*Otu 08877	*Xenasmatella vaga*	KP814432.1	6E-137	92	0.98	2.37	1.67
11	*Xylaria*Otu 10695	*Xylaria* sp.	KT291429.1	0	99	1.62	1.56	1.59
12	*Cortinarius*Otu 10689	n.a. coverage only 60%	n.a.	n.a.	n.a.	2.97	0	1.49
13	***Amauroderma*Otu 10686**	***Daedaleopsis* sp.**	KT334715.1	0	100	2.96	0	1.48
14	XylarialesOtu 10684	Xylariales	KU747727.1	7E-171	97	0.06	2.8	1.43
15	*Tinctoporellus*Otu 10682	*Tinctoporellus epimiltinus*	KJ654618.1	1E-173	99	0.05	2.44	1.25
16	*Phlebia*Otu 10688	*Phlebia* sp.	KJ654593.1	2E-167	98	1.89	0.13	1.01
17	*Pestalotiopsis*Otu 02571	*Pestalotiopsis* sp.	AB297793.1	6E-142	93	1.42	0.29	0.86
18	*Coprinopsis*Otu 09349	*Coprinopsis* sp.	AB509906.1	3E-179	100	0	1.64	0.82
19	*Dacrymyces*Otu 10712	Dacrymycetales	KJ714000.1	5E-128	92	0	1.42	0.71
20	*Penicillium*Otu 10681	*Penicillium lignorum*	NR_138299.1	6E-127	90	0.01	1.4	0.7

Table S5. Spearman's rank correlations (i) between wood decomposition rate and relative abundances of dominant wood-inhabiting fungal OTUs and (ii) among the relative abundances of dominant wood-inhabiting fungal OTUs in *Schima superba* deadwood (n = 57).

**Parameter**	**Wood decomposition rate**	***Xylaria* Otu 01638**	***Phanerochaete* Otu 03458**	**Sordariales Otu 04020**
**Wood decomposition rate**	0	0.000772	0.000196	0.024124
***Xylaria* Otu 01638**	-0.43281	0	0.018219	0.003212
***Phanerochaete* Otu 03458**	0.47388	-0.31181	0	0.000142
**Sordariales Otu 04020**	0.29847	-0.38373	0.48283	0

Table S6. Relative abundances of *Xylaria*Otu 01638 and *Phanerochaete*Otu 03458 detected in 38 *Schima superba* deadwood samples arrange according to increased wood decomposition rate (*k* rate). *Filled color in each cell indicates the occurrence patterns of each fungal OTU (orange = only XylariaOtu 01638 were detected; yellow = only *Phanerochaete*Otu 03458 were detected and green = both OTUs were co-occurred). Spearman's rank correlations (n = 38) were -0.76 (between relative abundances of *Xylaria*Otu 01638 and *Phanerochaete*Otu 03458), -0.61 (between relative abundances of *Xylaria*Otu and *k* rate) and 0.67 (between relative abundances of *Phanerochaete*Otu 03458 and *k* rate), all cases $P < 0.001$.

Sample	*k* rate	*Xylaria*	*Phanerochaete*
1	0.05	1.37	0.00
2	0.09	4.46	0.00
3	0.10	0.60	0.00
4	0.13	23.65	0.00
5	0.13	14.26	0.00
6	0.13	4.07	0.00
7	0.14	26.11	0.00
8	0.14	45.63	0.00
9	0.15	0.46	0.00
10	0.15	15.94	0.00
11	0.15	14.88	0.00
12	0.15	10.45	0.00
13	0.16	0.07	0.00
14	0.16	9.04	0.00
15	0.16	1.08	0.00
16	0.17	1.89	0.10
17	0.17	0.00	2.36
18	0.19	0.00	0.03
19	0.19	7.58	0.00
20	0.20	0.00	45.06
21	0.21	0.00	0.03
22	0.21	0.00	0.10
23	0.21	14.42	0.00
24	0.22	0.00	0.65
25	0.23	0.00	95.08
26	0.23	0.07	0.00
27	0.23	3.30	0.00
28	0.24	0.66	97.40
29	0.26	0.00	0.03
30	0.26	0.00	94.67
31	0.27	0.00	23.17
32	0.28	0.00	25.88
33	0.28	0.00	75.65
34	0.28	0.00	14.33
35	0.29	0.20	0.00
36	0.31	0.00	40.40
37	0.33	3.85	0.04
38	0.37	0.07	13.45

Figure S1. Individual rarefaction curves of wood-inhabiting fungi detected in each deadwood sample using dataset excluding rare OTUs (a) and full dataset (b).

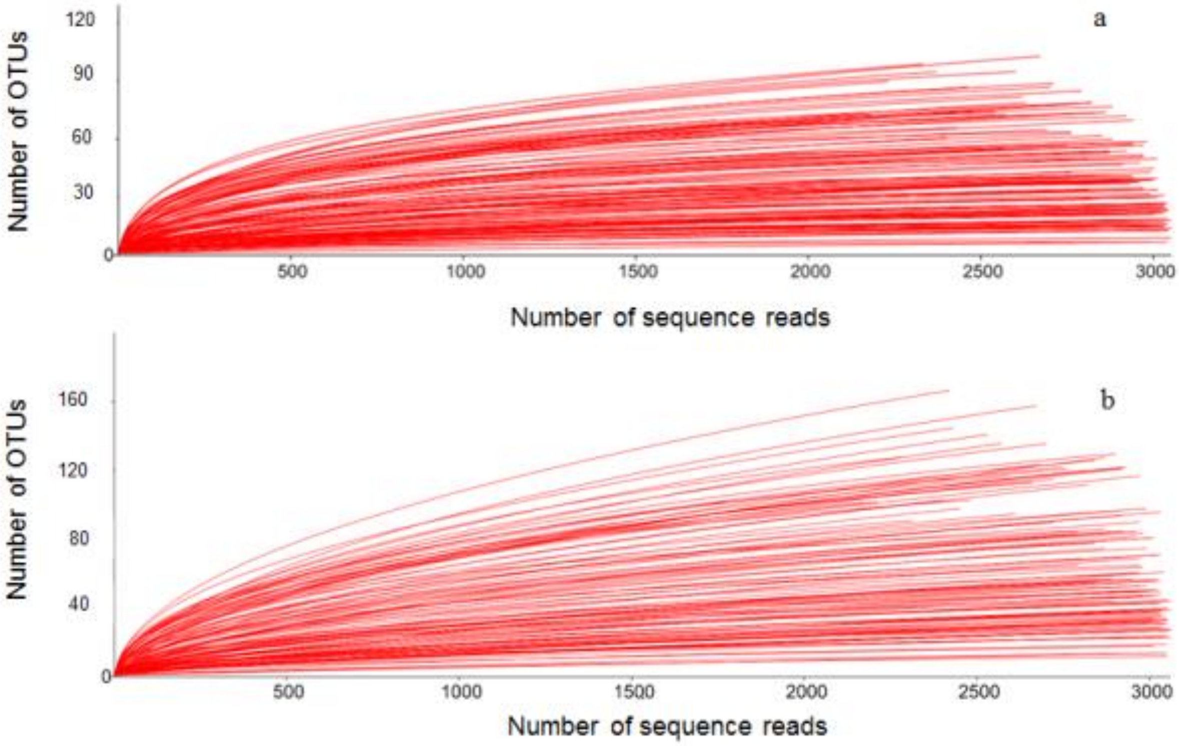

Figure S2. Wood-inhabiting fungal community composition associated with *Pinus massoniana* calculated using abundance (a) and presence-absence data (b). These charts show the proportion of OTUs assigned to each fungal phylum (mainly Ascomycota and Basidiomycota). NA = not assigned.

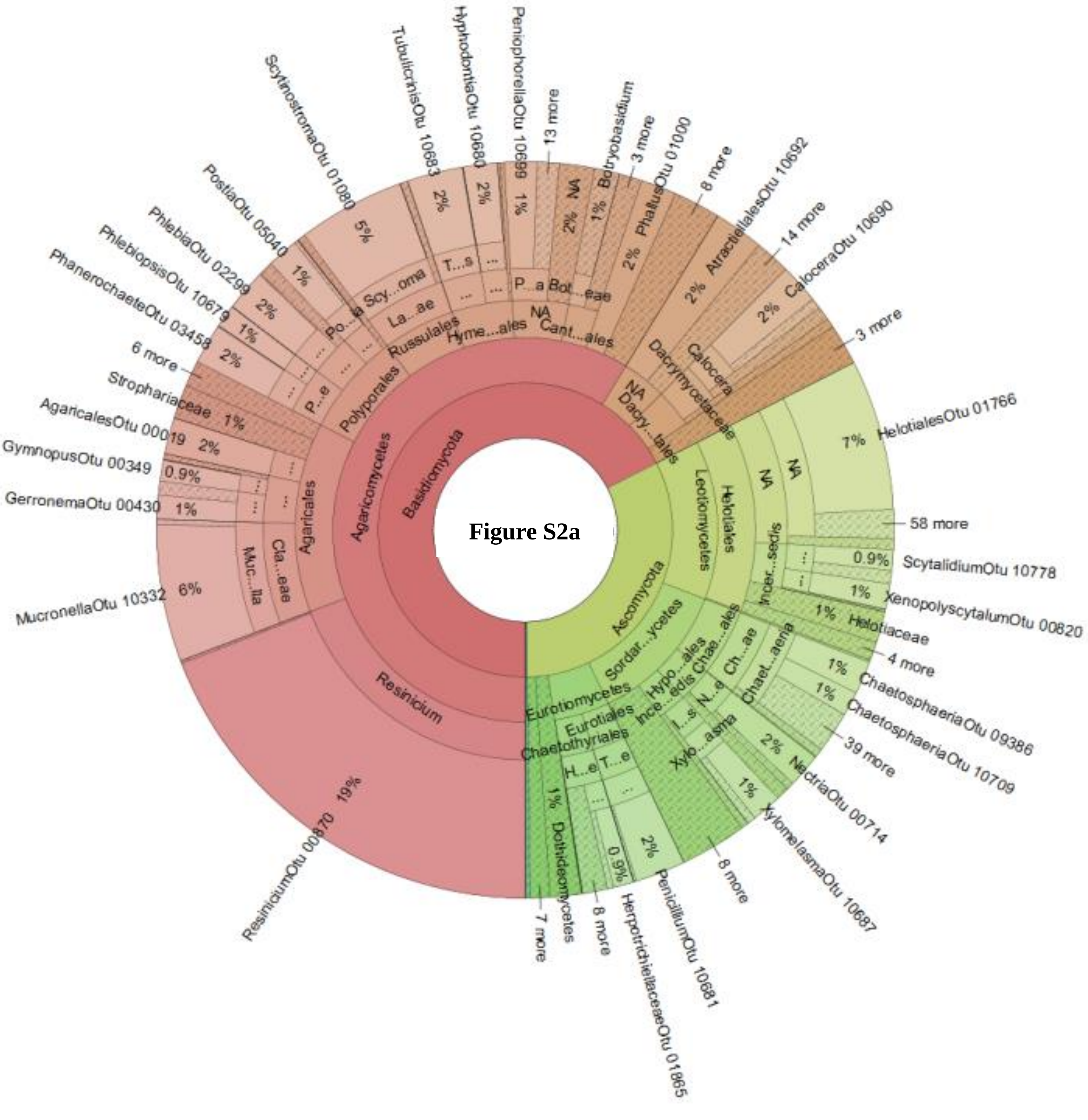

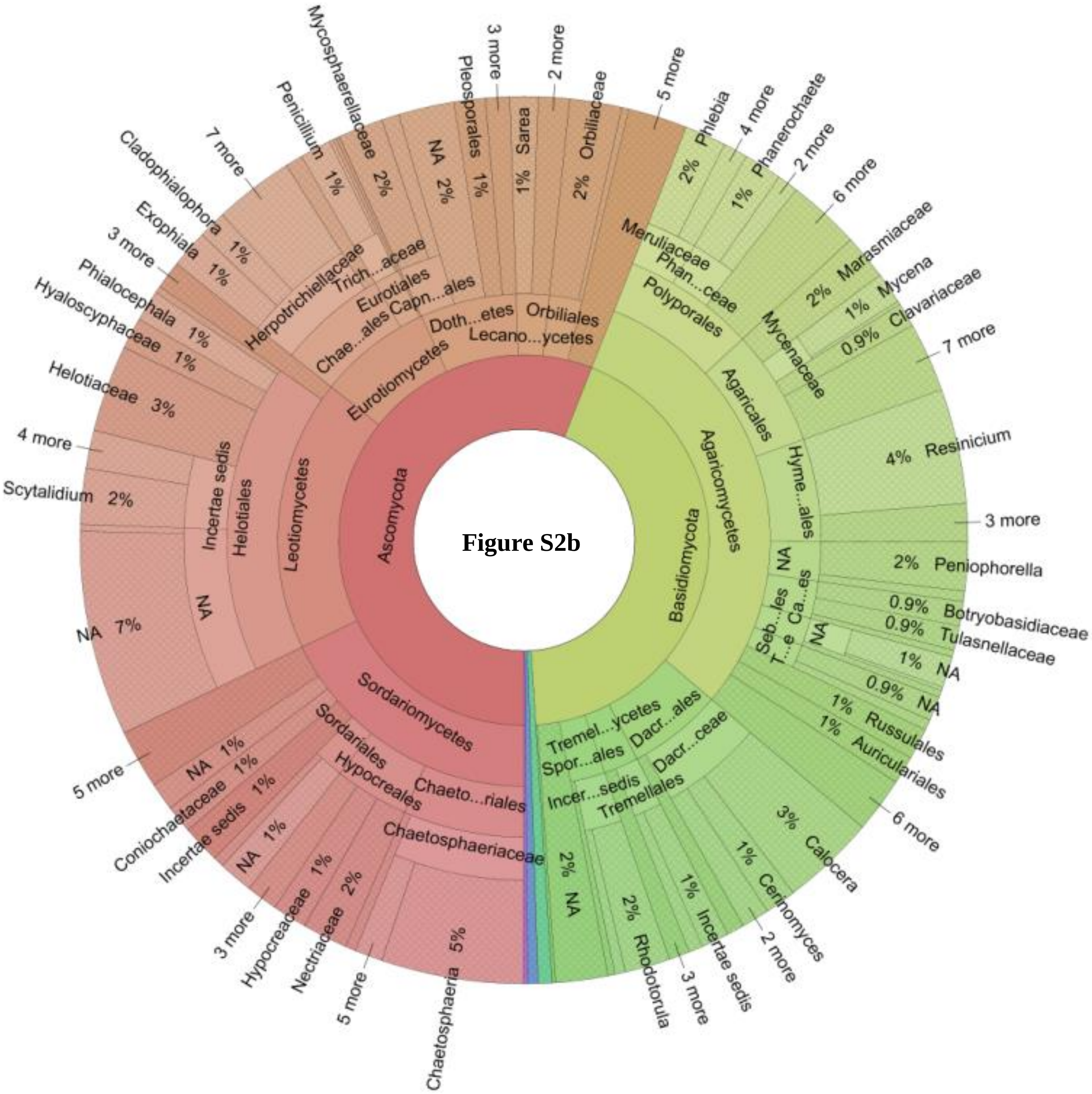
Ascomycota
Basidiomycota
Leotiomycetes
Eurotiomycetes
Sordariomycetes
Lecano...ycetes
Doth...etes
Orbiliales
Agaricomycetes
Tremel...ycetes
Helotiales
Incertae sedis
NA
Herpotrichiellaceae
Trich...aceae
Eurotiales
Capn...ales
Chae...ales
Sordariales
Hypocreales
Chaeto...riales
Chaetosphaeriaceae
Polyporales
Agaricales
Hyme...ales
Meruliaceae
Phan...ceae
Mycenaceae
Seb...les
T...e
Ca...es
Spor...ales
Dacr...ales
Dacr...ceae
Incer...sedis
Tremellales
Mycosphaerellaceae 2%
Penicillium 1%
Pleosporales 1%
NA 2%
3 more
2 more
Sarea 1%
Orbiliaceae 2%
5 more
Phlebia 2%
4 more
Phanerochaete 1%
2 more
6 more
Marasmiaceae 2%
Mycena 1%
Clavariaceae 0.9%
7 more
Resinicium 4%
3 more
Peniophorella 2%
Botryobasidiaceae 0.9%
Tulasnellaceae 0.9%
NA 1%
NA 0.9%
Russulales 1%
Auriculariales 1%
6 more
Calocera 3%
Cerinomyces 1%
2 more
Incertae sedis 1%
3 more
Rhodotorula 2%
NA 2%
Chaetosphaeria 5%
5 more
Nectriaceae 2%
Hypocreaceae 1%
3 more
NA 1%
Incertae sedis 1%
Coniochaetaceae 1%
NA 1%
5 more
NA 7%
Scytalidium 2%
4 more
Helotiaceae 3%
Hyaloscyphaceae 1%
Phialocephala 1%
3 more
Exophiala 1%
Cladophialophora 1%
7 more

Figure S2b

Figure S3. Wood-inhabiting fungal community composition associated with *Schima superba* calculated using abundance (a) and presence-absence data (b). These charts show the proportion of OTUs assigned to each fungal phylum (mainly Ascomycota and Basidiomycota). NA = not assigned.

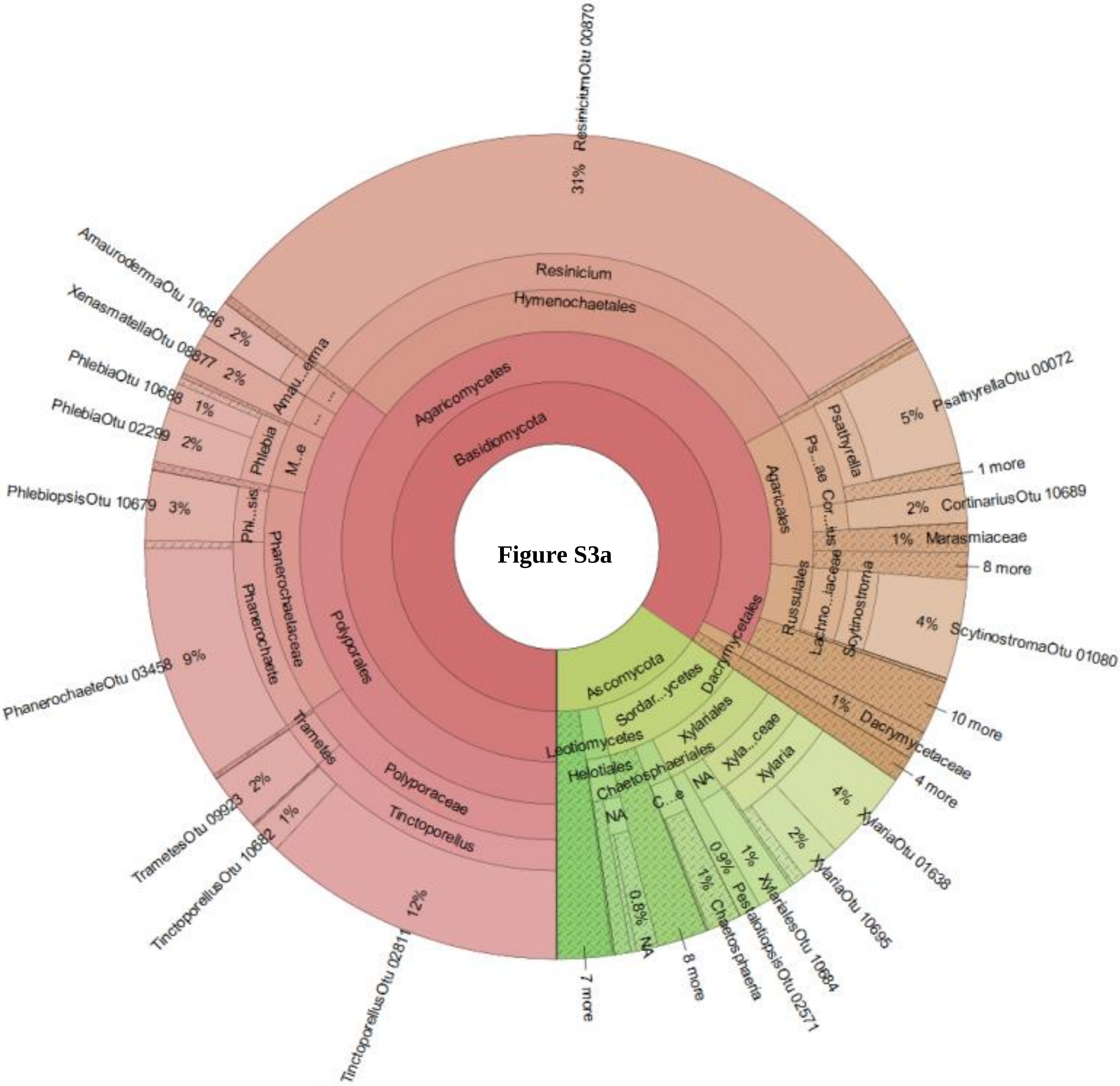

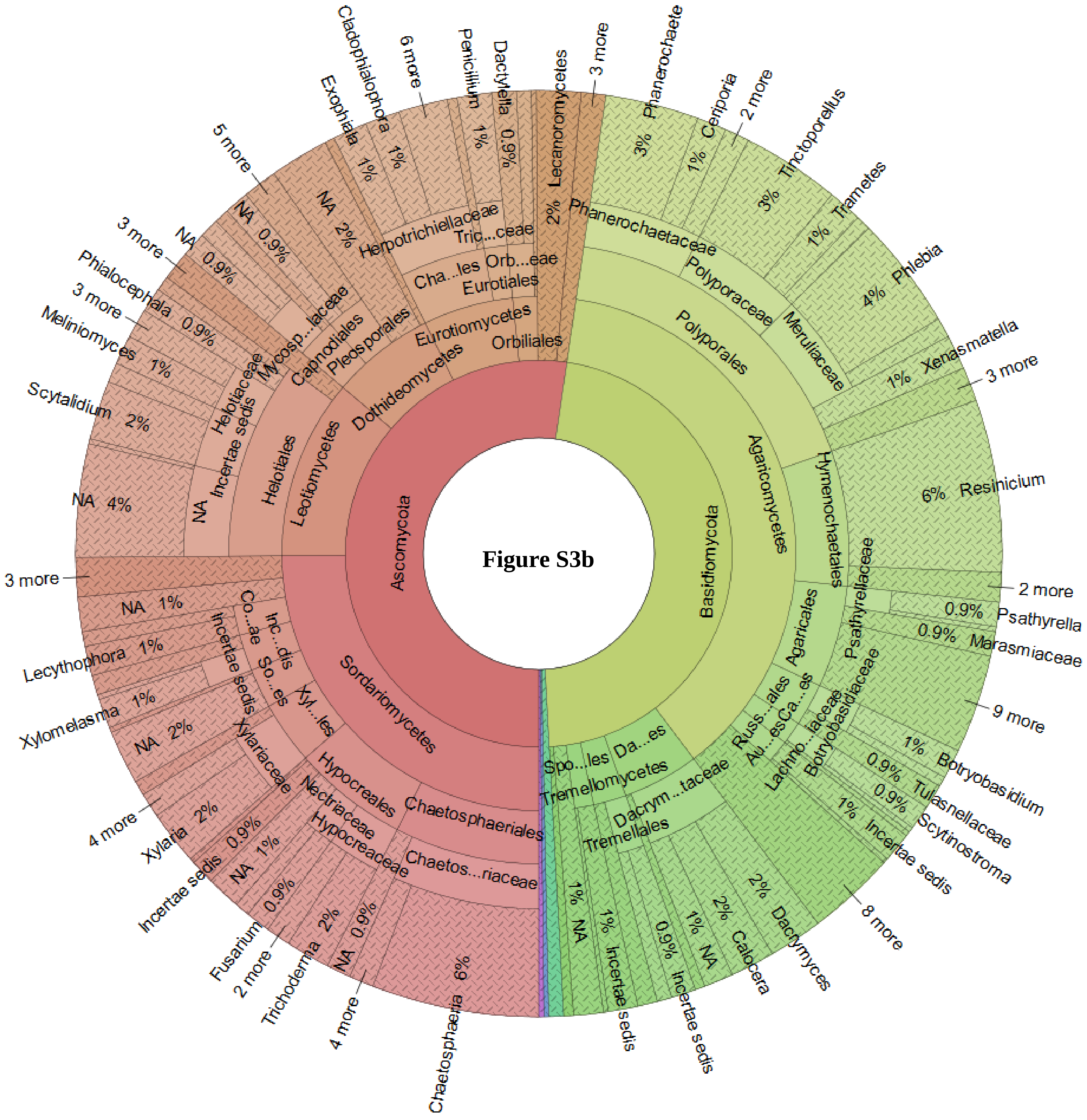
Figure S3b
Ascomycota
Basidiomycota
Dothideomycetes
Leotiomycetes
Eurotiomycetes
Orbiliales
Lecanoromycetes
Sordariomycetes
Agaricomycetes
Tremellomycetes
Eurotiales
Cha...les Orb...eae
Herpotrichiellaceae
Tric...ceae
Pleosporales
Capnodiales
Mycosp...laceae
Helotiales
Helotiaceae
Incertae sedis
Hypocreales
Chaetosphaeriales
Nectriaceae
Hypocreaceae
Chaetos...riaceae
Xylariaceae
Xyl...les
Inc...dis
Co...ae So...es
Polyporales
Polyporaceae
Meruliaceae
Phanerochaetaceae
Hymenochaetales
Agaricales
Psathyrellaceae
Botryobasidiaceae
Russ...ales
Au...esCa...es
Lachno...laceae
Dacrym...taceae
Tremellales
Spo...les Da...es
Cladophialophora 1%
Exophiala 1%
6 more
Penicillium 1%
Dactylella 0.9%
2%
3 more
5 more
NA 0.9%
NA 2%
3 more
NA 0.9%
Phialocephala 0.9%
3 more
Meliniomyces 1%
Scytalidium 2%
NA 4%
3 more
NA 1%
Lecythophora 1%
Xylomelasma 1%
NA 2%
4 more
Xylaria 2%
Incertae sedis 0.9%
NA 1%
Fusarium 0.9%
2 more
Trichoderma 2%
NA 0.9%
4 more
Chaetosphaeria 6%
Phanerochaete 3%
Ceriporia 1%
2 more
Tinctoporellus 3%
Trametes 1%
Phlebia 4%
Xenasmatella 1%
3 more
Resinicium 6%
2 more
Psathyrella 0.9%
Marasmiaceae 0.9%
9 more
Botryobasidium 1%
Tulasnellaceae 0.9%
Scytinostroma 0.9%
Incertae sedis 1%
8 more
Dacrymyces 2%
Calocera 2%
NA 1%
Incertae sedis 0.9%
Incertae sedis 1%
NA 1%

References

Bruelheide, H., Böhnke, M., Both, S., Fang, T., Assmann, T., Baruffol, M., et al. (2011). Community assembly during secondary forest succession in a Chinese subtropical forest. Ecol. Monogr. 81, 25–41. doi:10.1890/09-2172.1.

Gardes M, Bruns TD (1993) ITS primers with enhanced specificity for basidiomycetes-application to the identification of mycorrhizae and rusts. Mol Ecol 2:113–118.

Hoppe B, Purahong W, Wubet T, et al (2015) Linking molecular deadwood-inhabiting fungal diversity and community dynamics to ecosystem functions and processes in Central European forests. Fungal Divers 1–13. doi: 10.1007/s13225-015-0341-x

Lentendu G, Wubet T, Chatzinotas A, et al (2014) Effects of long-term differential fertilization on eukaryotic microbial communities in an arable soil: a multiple barcoding approach. Mol Ecol 23:3341–3355. doi: 10.1111/mec.12819

Kõljalg U, Nilsson RH, Abarenkov K, et al. (2013) Towards a unified paradigm for sequence-based identification of Fungi. Mol Ecol 22: 5271–5485

Kubartová A, Ottosson E, Dahlberg A, Stenlid J (2012) Patterns of fungal communities among and within decaying logs, revealed by 454 sequencing. Mol Ecol 21:4514–4532. doi: 10.1111/j.1365-294X.2012.05723.x

Ottosson E, Kubartová A, Edman M, et al (2015) Diverse ecological roles within fungal communities in decomposing logs of *Picea abies*. FEMS Microbiol Ecol 91:fiv012. doi: 10.1093/femsec/fiv012

Ovaskainen O, Schigel D, Ali-Kovero H, et al (2013) Combining high-throughput sequencing with fruit body surveys reveals contrasting life-history strategies in fungi. ISME J 7:1696–1709. doi: 10.1038/ismej.2013.61

Schuldt, A., Wubet, T., Buscot, F., Staab, M., Assmann, T., Böhnke-Kammerlander, M., et al. (2015). Multitrophic diversity in a biodiverse forest is highly nonlinear across spatial scales. Nat. Commun. 6, 10169. doi:10.1038/ncomms10169.

Staab, M., Schuldt, A., Assmann, T., Bruelheide, H., and Klein, A.-M. (2014). Ant community structure during forest succession in a subtropical forest in South-East China. Acta Oecologica 61, 32–40. doi:10.1016/j.actao.2014.10.003.

van der Wal A, Ottosson E, de Boer W (2014) Neglected role of fungal community composition in explaining variation in wood decay rates. Ecology 96:124–133. doi: 10.1890/14-0242.1

White TJ, Bruns TD, Lee S, Taylor J (1990) Amplification and Direct Sequencing of Fungal Ribosomal RNA Genes for Phylogenetics. In: Innis MA, Gelfand DH, Sninsky JJ, White TJ (eds). PCR Protocols: A Guide to Methods and Applications. Academic Press, San Diego, pp 315–322

Yamashita S, Masuya H, Abe S, et al (2015) Relationship between the decomposition process of coarse woody debris and fungal community structure as detected by high-throughput sequencing in a deciduous broad-leaved forest in Japan. PloS One 10:e0131510. doi: 10.1371/journal.pone.0131510
